# Statistical Evaluation of Different Mathematical Models for Diffusion Weighted Imaging of Prostate Cancer Xenografts in Mice

**DOI:** 10.3389/fonc.2021.583921

**Published:** 2021-05-26

**Authors:** Harri Merisaari, Hanne Laakso, Heidi Liljenbäck, Helena Virtanen, Hannu J. Aronen, Heikki Minn, Matti Poutanen, Anne Roivainen, Timo Liimatainen, Ivan Jambor

**Affiliations:** ^1^ Department of Radiology, University of Turku, Turku, Finland; ^2^ Turku Brain and Mind Center, University of Turku, Turku, Finland; ^3^ Department of Biotechnology and Molecular Medicine, A.I. Virtanen Institute for Molecular Sciences, Kuopio, Finland; ^4^ Turku PET Centre, University of Turku and Turku University Hospital, Turku, Finland; ^5^ Turku Center for Disease Modeling, University of Turku, Turku, Finland; ^6^ Medical Imaging Centre of Southwest Finland, Turku University Hospital, Turku, Finland; ^7^ Department of Oncology and Radiotherapy, Turku University Hospital, Turku, Finland; ^8^ Research Unit of Medical Imaging, Physics and Technology, University of Oulu, Oulu, Finland; ^9^ Department of Clinical Radiology, Oulu University Hospital, Oulu, Finland; ^10^ Department of Radiology, University of Oulu, Oulu, Finland

**Keywords:** diffusion weighted imaging, PC-3 xenograft prostate tumors, prostate cancer mouse model, repeatability, Akaike information criteria (AIC), F-ratio

## Abstract

**Purpose:**

To evaluate fitting quality and repeatability of four mathematical models for diffusion weighted imaging (DWI) during tumor progression in mouse xenograft model of prostate cancer.

**Methods:**

Human prostate cancer cells (PC-3) were implanted subcutaneously in right hind limbs of 11 immunodeficient mice. Tumor growth was followed by weekly DWI examinations using a 7T MR scanner. Additional DWI examination was performed after repositioning following the fourth DWI examination to evaluate short term repeatability. DWI was performed using 15 and 12 b-values in the ranges of 0-500 and 0-2000 s/mm^2^, respectively. Corrected Akaike information criteria and F-ratio were used to evaluate fitting quality of each model (mono-exponential, stretched exponential, kurtosis, and bi-exponential).

**Results:**

Significant changes were observed in DWI data during the tumor growth, indicated by ADC_m_, ADC_s_, and ADC_k_. Similar results were obtained using low as well as high b-values. No marked changes in model preference were present between the weeks 1−4. The parameters of the mono-exponential, stretched exponential, and kurtosis models had smaller confidence interval and coefficient of repeatability values than the parameters of the bi-exponential model.

**Conclusion:**

Stretched exponential and kurtosis models showed better fit to DWI data than the mono-exponential model and presented with good repeatability.

## Introduction

Diffusion weighed imaging (DWI) has extensively been used for cancer characterization in both pre-clinical (1, 2) and clinical settings (3) during the last decade. Furthermore, DWI is increasingly being used for monitoring cancer therapy responses (4). In biological tissue, DWI contrast is predominantly affected by microscopic motion of water molecules and water interactions with surroundings. The most recognized DWI imaging acquisition method is the Stejskal–Tanner pulsed field gradient method. With this method, motion caused by self diffusion of a proton is acquired by applying a pair of motion-encoding gradients. The first gradient dephases and second one rephrases stationary protons, while moving water protons stays dephased resulting to decreased signal intensity. The signal attenuation depends on water diffusion coefficient (D [mm^2^/s]) as well as direction of the self diffusion of water (5).

Several different mathematical models have been proposed to describe the DWI signal decay. The mono-exponential model is the simplest and widely used, in which one parameter D (or often the apparent diffusion coefficient, ADC) describes the diffusion. This model fits well to DWI data measured from pure water without any restrictions. At low b-values DWI signal decay deviates from the mono-exponential function due to presence of intra-voxel incoherent motion (IVIM), as originally proposed by Le Bihan and co-workers (6, 7). The search for new non-invasive imaging markers has led to increased interests in IVIM-derived parameters, which have demonstrated correlation with microvessel density in colorectal cancer (HT29) model (8). Nevertheless, IVIM-derived parameters are not directly related to tissue perfusion (6, 7), but perfusion and IVIM-derived parameters are rather related to the capillary structure (9, 10). Similarly to low b-values, DWI signal decay at high b-values deviates from the mono-exponential function, and it is better described by non-Gaussian mathematical models (11–14).

In general, a single mono-exponential decay provides oversimplified description of the complicated water motion in the tissue. However, the modeling with several free parameters could lead to “over-fitting” of data, and poor repeatability of the fitted parameters. Optimal model would have the highest information content and provides independent parameters, which are related to physical quantities (e.g. cell and/or vessel density) while still retain high repeatability/reliability of fitted parameters.

The Akaike information criteria (AIC) has been widely used for model selection in previous studies (15–17). A model with smaller AIC value would be a preferred model due to less information loss as compared with a model presenting with higher AIC. Similarly to AIC, F-ratio is commonly being applied for model selection (18). Model selection based on F-ratio tends to prefer a more simplified model (19) in contrast to AIC.

In the current study, we evaluated four different mathematical models for DWI within a study applying PC-3 prostate cancer cells grown in immunodeficient mice using both low (0-500 s/mm^2^) and high b-values (0-2000 s/mm^2^). The tumor growth was followed for four weeks with repeated MR examinations performed once a week. Corrected Akaike information criteria (AIC_c_) and F-ratio were used to evaluate information content of the models. Non-Gaussian DWI models provided better fit to DWI data obtained using both low and high b-values. However, non-Gaussian DWI models were not clearly preferred over the mono-exponential model for DWI data obtained using low b-values in contrast to DWI data obtained using high b-values. Furthermore, DWI fitted parameters changed significantly during tumor progression.

## Material and Methods

### Animal Tumor Model

One million PC-3 (Anticancer Inc., USA) human prostate cancer cells were inoculated subcutaneously in immunodeficient mice (n=11, HSD: Athymic Nude Foxn 1nu, Harlan Laboratories, Indianapolis, IN, USA). The cells also expressed red florescent protein, while this property was not applied in the present study. Mice were housed in individually ventilated cages under controlled conditions of light (12h light/12h dark), temperature (21 ± 3°C), and humidity (55% ± 15%) in specific pathogen-free conditions at the Central Animal Laboratory, University of Turku for the first 5 days, and thereafter in similar conditions at the University of Eastern Finland Kuopio campus. Mice were provided with irradiated soy-free natural-ingredient feed (RM3 (E), Special Diets Services, Essex, UK) and autoclaved tap water *ad libitum*, and were housed complying with international guidelines on the care and use of laboratory animals. All animal handling was conducted in accordance with the Finnish Committee for the use and care of laboratory animals and the institutional animal care policies, which fully meet the requirements as defined in the U.S. National Institutes of Health guidelines on animal experimentation.

### MR Imaging

The first MR examination was performed 8 days after cell implantation. Tumor growth was followed for four weeks with repeated MR examinations once a week. Immediately following the fourth MR examination, six and seven mice had repeated DWI scan performed using low and high b-values, respectively. The second repetition was performed following animal and coil repositioning approximately 60 minutes after the first set of DWI. The repeated DWI examinations were used to evaluate short term repeatability of the measured parameters. The anesthetized mice (1.5% isoflurane in 70%N_2_/30%O_2_) were imaged using a 7T animal MR scanner (7T Pharmascan, Bruker GmbH, Ettlingen, Germany) with 72 mm volume transmitter (Bruker GmbH) and 10 mm surface receiver coil (Bruker GmbH). Multislice T_2_-weighted anatomical images covering the whole tumor area were obtained (TR/TE 2500 ms/33 ms, field of view (FOV) = 30 × 30 mm^2^, matrix size 256 × 256, 15 slices) to localize a slice with maximum tumor diameter for DWI measurements. Diffusion weighted single shot spin-echo echo planar imaging was applied with the parameters: TR/TE 3750/25.3 (low b-value set) 3000/30 ms (high b-value set), FOV 3 × 1.5 cm^2^, matrix 128 ×64, slice thickness 1 mm, three orthogonal diffusion directions, and two different sets of b-values: low b-value set (15 b-values in total): 0, 2, 4, 6, 9, 12, 14, 18, 23, 25, 28, 50, 100, 300, 500 s/mm^2^, and high b-value set (12 b-values in total): 0, 100, 300, 500, 700, 900, 1100, 1300, 1500, 1700, 1900, 2000 s/mm^2^. For further analysis, the mean value of the signal from three directions was calculated.

### Data Modeling

The following four mathematical models were applied to the DWI signal obtained using low and high b-values:

1. Mono-exponential model:

[1] $$S(b)=S(0)e^{-bADC_{m}}$$

where b is the b-value, S(0) is the signal intensity at b-value of 0 s/mm^2^, and ADC_m_ is the apparent diffusion coefficient calculated using the mono-exponential model.

2. Stretched exponential model also known as Kohlrausch-Williams-Watts model (20):

[2] $$S(b)=S(0)e^{-{\left(bADC_{s}\right)}^{\alpha }}$$

where ADC_s_ is the apparent diffusion coefficient calculated using the stretched exponential model, and α is the heterogeneity index. The dimensionless α parameter varies from 0 to 1. During the fitting procedure, α parameter was constrained to be in the range of 0 to 1.

3. Kurtosis model:

[3] $$S(b)=S(0)e^{\left(-bADC_{k}+\frac{1}{6}b^{2}ADC_{k}^{2}K\right)}$$

where ADC_k_ is the apparent diffusion coefficient calculated using the kurtosis model, and K is the kurtosis. Jensen at al. (21) originally developed the kurtosis model to fit deviation of diffusion tensor signal from the mono-exponential function. The dimensionless positive K parameter characterizes the deviation from the mono-exponential signal decay.

4a. Bi-exponential model for low b-values:

[4] $$S(b)=S(0)(1-f_{p})e^{-bD_{f}}+f_{p}e^{-bD_{p}}$$

where f_p_ is the “pseudodiffusion” fraction, D_f_ is the fast diffusion coefficient, and D_p_ is the “pseudodiffusion” coefficient. The intravoxel incoherent motion (IVIM) theory is an advanced method to separate diffusion and perfusion effects using DWI (6) at low b-values. According to the IVIM theory, the blood flow in the capillaries causes a dephasing of the magnetization when motion-encoding gradients are applied. This means that the motion of water molecules due to microcirculation of blood in the capillaries has a similar effect on the resulting DWI signal as their motion due to molecular diffusion.

4b. Bi-exponential model for high b-values:

[5] $$S(b)=S(0)(1-f_{f})e^{-bD_{s}}+f_{f}e^{-bD_{f}}$$

where f_f_ is the fraction of fast diffusion, D_f_ is the fast diffusion coefficient, and D_s_ is the slow diffusion coefficient.

The DWI signal decay of each individual voxel has been fitted using four mathematical models, as described above, to generate parametric maps of the parameters. The fitting procedure has been performed using in-house written C++ code utilizing Broyden–Fletcher–Goldfarb–Shanno (BFGS) algorithm (22) in dlib library (23).

Following multiple initializations values were used to prevent local minima in the fitting procedure in order to avoid local minima in the fitting procedure (initializations values for high b-values data are in brackets):

1. Mono-exponential:

$ADC_{m}–\;\text{from}\;0.001\;\left(0.001\right)\;\text{to}\;0.003\;\left(0.003\right)\;\text{with}\;\text{the}\;\text{step}\;\text{size}\;\text{of}\;0.0005\;\left(0.0005\right)$

2. Stretched exponential:

$ADC_{s}–\;\text{from}\;0.001\;\left(0.001\right)\;\text{to}\;0.003\;\left(0.003\right)\;\text{with}\;\text{the}\;\text{step}\;\text{size}\;\text{of}\;0.0005\;\left(0.0005\right)$

α–from 0.1 (0.1) to 1.0 (1.0) with the step size of 0.15 (0.15)

3. Kurtosis:

$ADC_{k}–\;\mathrm{from}\;0.001\;\text{to}\;0.003\;\text{with}\;\text{the}\;\text{step}\;\text{size}\;\text{of}\;0.0003$

K–from 0.0001 (0.0001) to 4.0 (2.0) with the step size of 0.05 (0.02)

4a (b). Biexponential:

$f_{p}\left(f_{f}\right)–\text{from}\;0.0\;\left(0.5\right)\;\text{to}\;1.0\;\left(1.0\right)\;\text{with}\;\text{the}\;\text{step}\;\text{size}\;\text{of}\;\;0.1\;\left(0.1\right)$

$D_{p}\left(D_{f}\right)–\text{from}\;0.0001\;\left(0.0001\right)\;\text{to}\;0.04\;\left(0.003\right)\;\text{with}\;\text{the}\;\text{step}\;\text{size}\;\text{of}\;\;0.0005\;\left(0.0003\right)$

$D_{f}\left(D_{s}\right)–\text{from}\;0.0001\;\left(0.00002\right)\;to\;0.003\;\left(0.001\right)\;\text{with}\;\text{the}\;\text{step}\;\text{size}\;\text{of}\;\;0.0003\;\left(0.00005\right)$

### Image Analysis

The tumor area was manually delineated on T2-weighted anatomical images and the regions of interest (ROIs) were defined to the corresponding parametric images. Voxels with ADC_m_ values higher than 8.0^10^-3^ s^2^/mm were discarded as those voxels were considered to represent necrosis. Median values of the fitted parameters of each ROI between repeated scans were compared using one-way analysis of variance with Bonferroni test (p<0.05 statistically significant).

## Evaluation of Fitting Quality

Corrected Akaike information criteria difference (ΔAIC_c_) (15) was used to evaluate model fit to DWI data of each individual voxel:

[6] $$\Delta AIC_{c}=N\left(\ln(\frac{SS_{B}}{N})-\ln\left(\frac{SS_{A}}{N}\right)\right)+2(P_{B}-P_{A})+2\left(\frac{P_{B}(P_{B}+1)}{N-P_{B}-1}-\frac{P_{A}(P_{A}+1)}{N-P_{A}-1}\right)$$

where N is the sample size, P is the number of parameters, SS is the sum of squares between data points and fitted curve, A subscript represents the simpler model, B subscripts represents the more complex model.

In addition to ΔAIC_c_, F-ratio (F) with 1% level of significance was used to evaluate model fit to data:

[7] $$F=\frac{(SS_{A}-SS_{B})/SS_{B}}{(DF_{A}-DF_{B})/DF_{B}}$$

where DF (= number of data points − number of parameters) is the degree of freedom, A subscript represents the simpler model, B subscripts represents the more complex model.

## Repeatability of the Fitted Parameters

Repeatability of the fitted parameters was evaluated using the same methodology as in a previous human DWI drug intervention study (24). The difference (d) in median values per ROI between two repeated scans performed 4 weeks after the initial scan was calculated for a subset of mice (six mice for low b-values and seven for high b-values). Mean squared difference (msd) was calculated as follows:

[8] $$msd=\sqrt{\sum_{i=1}^{n}d^{2}\times {\left(n-1\right)}^{-1}}$$

where d is the difference between two repeated scans, n is the number of mice with repeated scan. Subsequently, 95% confidence interval (CI) for changes in the study cohort was calculated:

[9] $$CI=\pm 1.96\;\times msd/\sqrt{n}$$

where msd is the mean squared difference, n is the number of mice with repeated scan.Finally, coefficient of repeatability (r) was calculated as follows:

[10] r=1.96 ×msd

## Results

PC-3 cancer cell growth was followed for 4 weeks with repeated MR examinations performed once a week. The changes in diffusion parameters are visualized in parametric maps on top of T2-weighted images for a representative case (**Figures 1** and **2**) while the rest of data are shown in Supplementary material (**Figures S1**–**S20**). Median signal intensity of tumor ROI and the correcting fitted curves of week 1 and week 4 are shown in **Figure 3**. The data is shown for the same representative tumor as shown in **Figure 1**.

**Figure 1:**
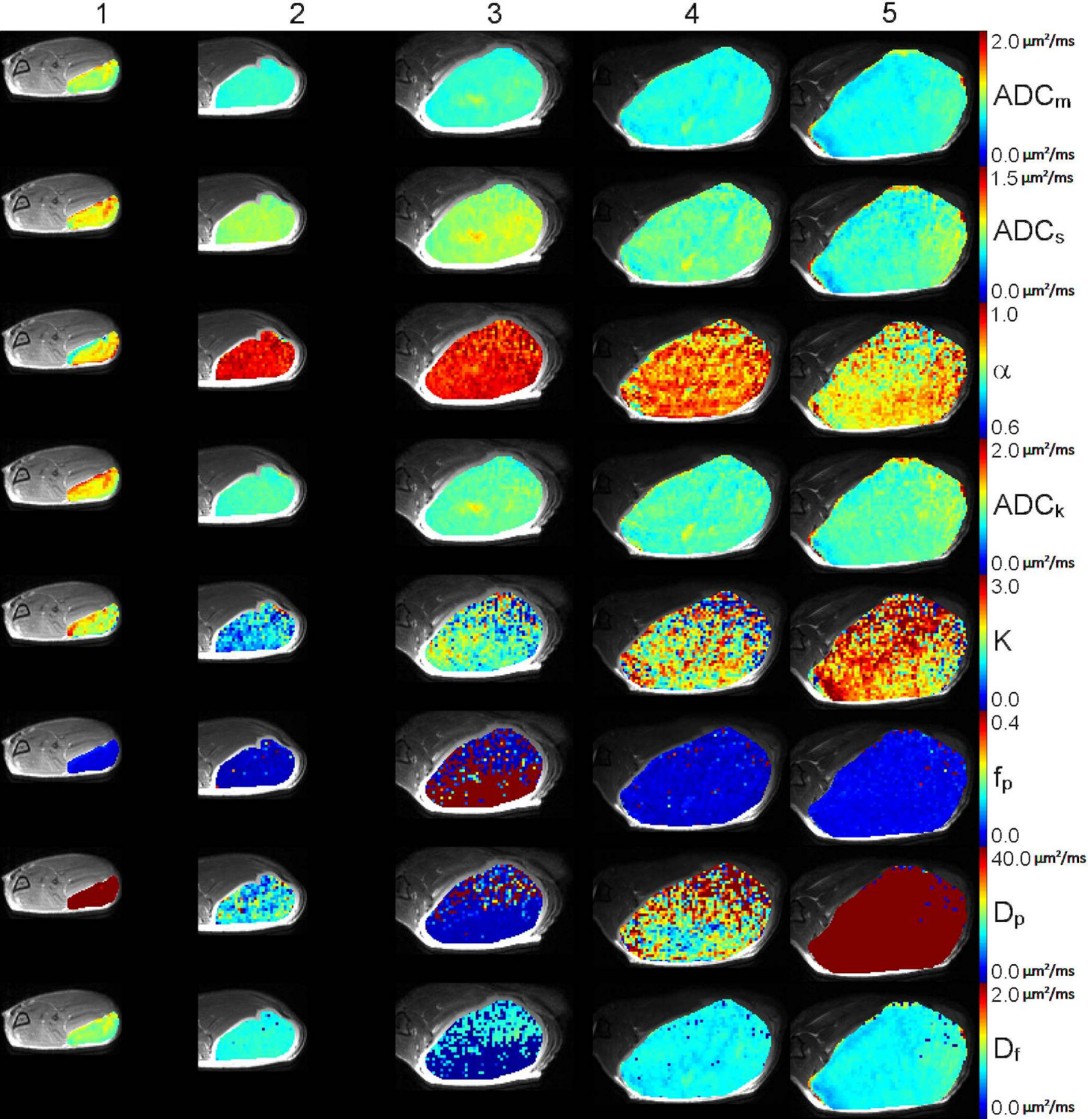
Low b-value DWI data of a representative tumor: T2-weighted image fused with parametric maps for ADC_m,_ ADC_s_, α, ADC_k_, K, f_p_, D_p_, and D_f_ parameters are shown, and represent different degree of tumor homogeneity between week 1 (column 1), week 2 (column 2), week 3 (column 4) and week 4 (column 1). Furthermore, the second repeated imaging on week 4 is shown (column 5). The parametric maps are scaled as follows: ADC_m,_ min−max: 0−2.0 µm^2^/ms; ADC_s,_ min−max: 0−1.5 µm^2^/ms; α, min−max: 0.6−1.0; ADC_k_, min−max: 0−2.0 µm^2^/ms; K, min−max: 0−3.0; f_p_, min−max: 0−0.4; D_p_, min−max: 0−40.0 µm^2^/ms; D_f_, min−max: 0−2.0 µm^2^/ms.

**Figure 2:**
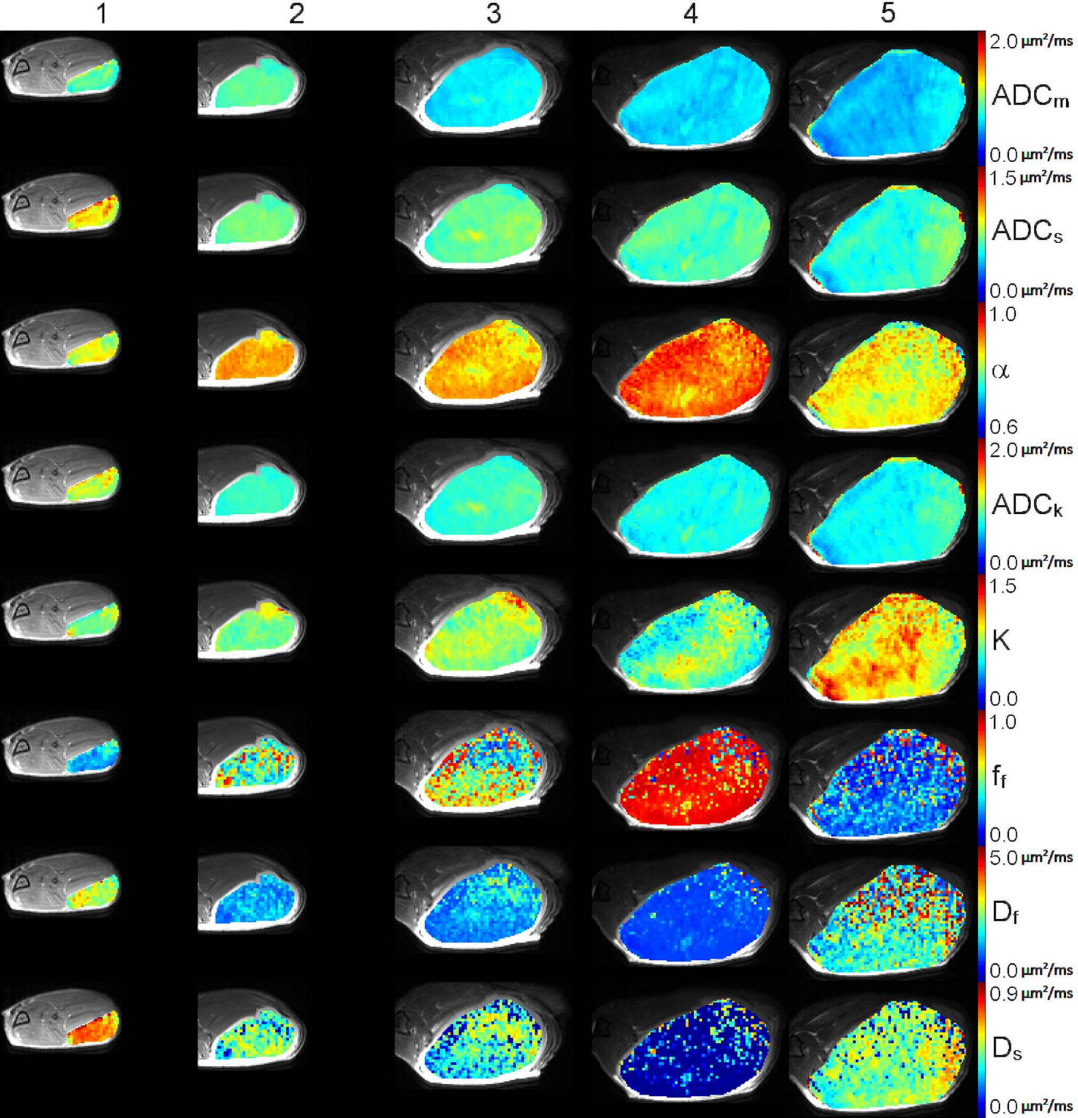
High b-value DWI data of a representative tumor: T2-weighted image fused with parametric maps for ADC_m,_ ADC_s_, α, ADC_k_, K, f_f_, D_f_, and D_s_ parameters are shown, and represent different degree of tumor homogeneity between week 1 (column 1), week 2 (column 2), week 3 (column 3), week 4 (column 4). Furthermore, the second repetition done on week 4 is shown (column 5). The parametric maps are scaled as follows: ADC_m_ (A1−5) min−max: 0−2.0 µm^2^/ms, ADC_s_ (B1−5) min−max: 0−1.5 µm^2^/ms, α (C1-5) min−max: 0.6−1.0, ADC_k_ (D1−5) min−max: 0−2.0 µm^2^/ms, K (E1−5), min−max: 0−1.5, f_f_ (F1−5), min−max: 0−1.0, D_f_ (G1−5) min−max: 0−5.0 µm^2^/ms, D_s_ (H1−5) min−max: 0−0.9 µm^2^/ms.

**Figure 3:**
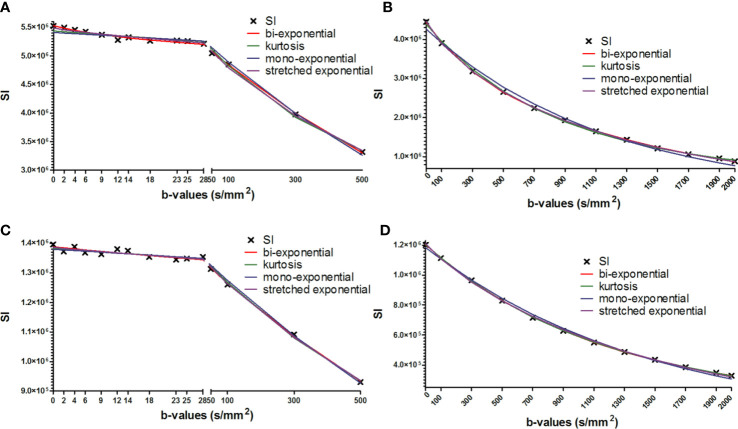
Mean signal intensity as a function of b-values (x-axis) fitted using all four models. The data is shown for the same representative tumor as shown in Figures 1 and 2. **(A)**; week 1, low b-value DWI data. **(B)**; week 1, high b-value DWI data. **(C)**; week 4, low b-value DWI data. **(D)**; week 4 high b-value DWI data. Bi-exponential, kurtosis and stretched exponential models provide better fit to the DWI decay curve than the mono-exponential model especially at high b-value DWI data.

**Figure 4:**
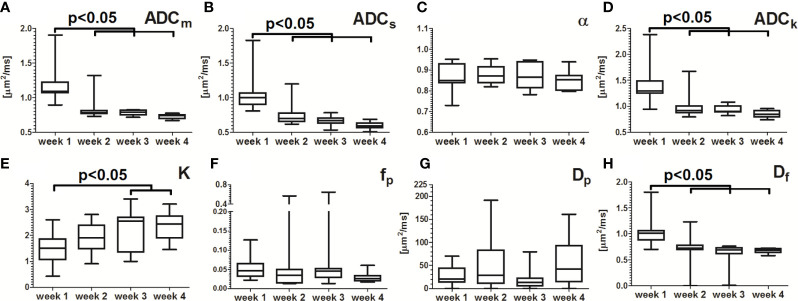
Median values of regions of interest (n=11) derived from DWI data obtained using low b-values. Significant changes (p<0.05) were present in ADC_m_ (part **A**), ADC_s_ (part **B**), ADC_k_ (part **D**), and D_f_ (part **H**) values between week 1 and those from the weeks 2, 3 and 4. K (part **E**) parameter differed significantly (p<0.05) between week 1 and weeks 3 and 4. The differences between weeks 2, 3 and 4 were not significant. The remaining differences in the fitted values (alpha, part **C**; Fp, part **F**; Dp part **G**) between weeks did not reach the level of statistical significance. The box extends from the 25^th^ to 75^th^ percentiles while the error bars extend from minimal to maximal values.

**Figure 5:**
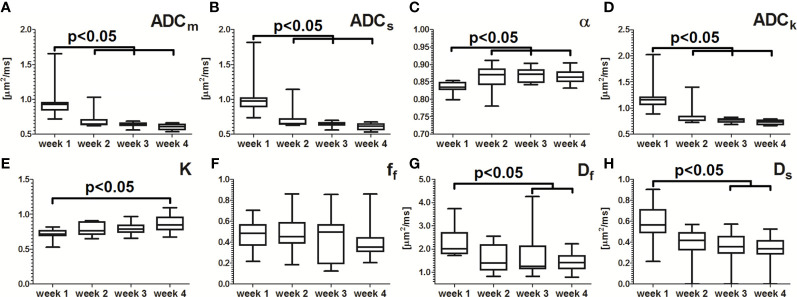
Median values of regions of interest (n=11) derived from DWI data obtained using high b-values. Significant changes (p<0.05) were present in ADC_m_ (part **A**), ADC_s_ (part **B**), alpha (part **C**), and ADC_k_ (part **D**) values between week 1 and weeks 2, 3 and 4. K (part **E**) parameter differed significantly (p<0.05) between week 1 and week 4. The differences between weeks 2, 3 and 4 were not significant. Values of D_f_ (part **G**) and D_s_ (part **H**) parameters differed significantly between week 1 and weeks 3 and 4. The remaining differences in the fitted values between weeks did not reach the level of statistical significance (Ff, part **F**). The box extends from the 25^th^ to 75^th^ percentiles while the error bars extend from minimal to maximal values.

Median values of the fitted parameters ADC_m_, ADC_s_, ADC_k_, and D_s_ between week 1 and those measured at weeks 2, 3 and 4 differed significantly while the differences between weeks 2, 3 and 4 were not significant using the low b-value data. Similarly, median values of K parameter increased significantly between week 1 and week 3 and 4. In contrast, no significant differences were present between median values of different weeks for α, D_p_, and f_f_ parameters (**Figure 4**). Using high b-values, significant changes were present in median values of ADC_m_, ADC_s_, α, and ADC_k_ between week 1 and weeks 2, 3 and 4, while differences between week 2, 3 and 4 were not significant. The changes in K parameter were significant only between week 1 and week 4, while differences between week 1 and weeks 3 and 4 were significant for D_f_ and D_s_ parameters (**Figure 5**).

### Model Selection

In general, DWI data obtained using low b-values fitted better by the stretched exponential model as compared with mono-exponential, kurtosis and bi-exponential models based on AIC_c_ and F-ratio. In more than 50% of voxels the kurtosis model had lower AIC_c_ values than the mono-exponential model. However, the kurtosis model did not provide significantly better fit to data than the mono-exponential model in more than 50% of voxels (averaged medians of 11 mice) based on F-test. Similarly, the bi-exponential model was not preferred over mono-exponential in more than 50% of voxels (averaged medians of 11 mice). No dramatic changes in model preference were present between different time points (**Tables 1** and **2**).

**Table 1:** Mean ± standard deviation of median percentage values per mouse described better by the first model of the comparison is shown in the table for DWI data obtained using low b-values.

Low b-values		week 1*	week 2	week 3	week 4
AIC_c_	stretched *vs.* mono-ex	81 ± 29%	69 ± 32%	71 ± 30%	78 ± 28%
	kurtosis *vs.* mono-ex	79 ± 30%	59 ± 38%	62 ± 36%	51 ± 33%
	bi-ex *vs.* mono-ex	75 ± 33%	56 ± 41%	53 ± 38%	69 ± 31%
	kurtosis *vs.* stretched	12 ± 16%	15 ± 28%	19 ± 24%	9 ± 15%
	bi-ex *vs.* stretched	23 ± 31%	15 ± 18%	10 ± 23%	29 ± 35%
	bi-ex *vs.* kurtosis	61 ± 33%	49 ± 37%	41 ± 38%	63 ± 35%
F-ratio	stretched *vs.* mono-ex	70 ± 34%	45 ± 44%	38 ± 39%	36 ± 30%
	kurtosis *vs.* mono-ex	45 ± 35%	28 ± 40%	19 ± 26%	8 ± 19%
	bi-ex *vs.* mono-ex	64 ± 34%	39 ± 40%	35 ± 35%	16 ± 16%
	biex *vs*. stretched	6 ± 12%	2 ± 5%	0 ± 0%	12 ± 30%
	bi-ex *vs.* kurtosis	42 ± 32%	23 ± 28%	13 ± 17%	26 ± 35%

AIC_c_, Corrected Akaike information criteria; mono-ex, mono-exponential; bi-ex, bi-exponential; *tumor growth after subcutaneous inoculation of PC-3 prostate cancer cells in mice.

**Table 2:** Mean ± standard deviation of median percentage values per mouse described better by the first model of the comparison is shown in the table for DWI data obtained using high b-values.

High b-values		week 1*	week 2	week 3	week 4
AIC_c_	stretched *vs.* mono-ex	99 ± 4%	100 ± 1%	100 ± 1%	99 ± 2%
	kurtosis *vs*. mono-ex	99 ± 4%	100 ± 1%	100 ± 1%	100 ± 1%
	bi-ex *vs.* mono-ex	98 ± 8%	100 ± 2%	100 ± 1%	99 ± 2%
	kurtosis *vs.* stretched	69 ± 36%	81 ± 31%	71 ± 39%	78 ± 24%
	bi-ex *vs.* stretched	66 ± 38%	76 ± 27%	59 ± 39%	62 ± 31%
	bi-ex *vs.* kurtosis	36 ± 27%	33 ± 34%	18 ± 23%	16 ± 19%
F-ratio	stretched *vs.* mono-ex	96 ± 14%	95 ± 16%	94 ± 19%	94 ± 12%
	kurtosis *vs.* mono-ex	97 ± 10%	99 ± 3%	99 ± 2%	97 ± 4%
	bi-ex *vs.* mono-ex	100 ± 0%	100 ± 0%	100 ± 0%	100 ± 0%
	bi-ex *vs.* stretched	82 ± 32%	95 ± 8%	85 ± 27%	89 ± 18%
	bi-ex *vs.* kurtosis	92 ± 10%	92 ± 9%	93 ± 9%	94 ± 6%

AIC_c_, Corrected Akaike information criteria; mono-ex, mono-exponential; bi-ex, bi-exponential: *tumor growth after subcutaneous inoculation of PC-3 prostate cancer cells in mice.

In contrast to low b-values, in vast majority of voxels stretched exponential, kurtosis and bi-exponential models fitted DWI data obtained using high b-values better than the mono-exponential model based on AIC_c_ and F-test. The kurtosis model was preferred over the stretched exponential model in average in ~75% of voxels based on AIC_c_. The bi-exponential models still provided significantly better fit to data than the stretched exponential and kurtosis models based on F-test.

### Repeatability of the Fitted Parameters

The parameters of mono-exponential, stretched exponential and kurtosis models had confidence interval values smaller than 25% of the corresponding averaged median values (**Table 3**) for DWI data obtained using both low as well as high b-values. Similarly, coefficients of repeatability were smaller than 45% of the corresponding averaged median values (**Table 3**), with the exception of K parameter for low b-value DWI data (r% 59.7%). In contrast, the parameters of the bi-exponential model had much larger confidence interval and coefficient of repeatability values, especially for low b-value DWI data. Large confidence interval and coefficient of repeatability values for the parameters of bi-exponential model implicate poor measurement repeatability. Confidence interval and coefficient of repeatability values for mono-exponential, stretched exponential and kurtosis models we similar for DWI data acquired using low and high b-values. However, K parameter of kurtosis models had approximately 2-times higher relative coefficient of repeatability values than the ADC_m_, ADC_s_, ADC_k_ parameters for low as well as high b-values.

**Table 3:** Repeatability of the fitted parameters for DWI data obtained using low and high b-values.

	Low b-values			High b-values		
	avg median	95% CI (%)	r% (%)	avg median	95% CI (%)	r% (%)
**ADC_m_**	0.709^10^−3^ mm^2^/s	7.2	19.9	0.581^10^−3^ mm^2^/s	10.8	28.6
**ADC_s_**	0.586^10^−3^ mm^2^/s	11.7	28.8	0.583^10^−3^ mm^2^/s	11.8	31.4
**α**	0.86	4.9	11.9	0.87	3.9	10.4
**ADC_k_**	0.837^10^−3^ mm^2^/s	9.2	22.6	0.704^10^−3^ mm^2^/s	10.4	27.6
**K**	2.48	24.4	59.7	0.91	16.1	42.7
**f_p_**	0.09	200	491.8	NA	NA	NA
**f_f_**	NA	NA	NA	0.47	74.6	197.4
**D_p_**	74.819^10^−3^ mm^2^/s	162.9	399.1	NA	NA	NA
**D_f_**	0.544^10^−3^ mm^2^/s	58.9	163.2	1.564^10^−3^ mm^2^/s	62.2	164.5
**D_s_**	NA	NA	NA	0.271^10^−3^ mm^2^/s	96	254.8

avg median, averaged median values per mouse; 95% CI (%), 95% confidence interval expressed as percentage of the averaged median values per mouse (avg median); r% (%), coefficient of repeatability as percentage of the averaged median value per mouse (avg median); NA, not applicable.

Signal intensities at the second repeated DWI examination differed systematically from those measured at the first DWI examination, and the median values of ADC_m_, ADC_s_, and ADC_k_ parameters were lower in the repeated DWI in all mice.

## Discussion

The use of DWI for cancer detection, characterization and cancer therapy response monitoring continues to increase in both pre-clinical and clinical settings. Despite wide use of DWI, accurate and robust modeling of DWI signal decay remains a challenge. In the current study, we have evaluated four different mathematical models for DWI data (low and high b-values) of PC-3-cell derived human prostate cancer xenografts in mice, in terms of fitting quality and repeatability. Significant changes were observed in median values of ADC parameters detected between week 1 and those measured 1-3 weeks later, while the difference between the values obtained at weeks 2-4 were not significant. In previous studies cell density was shown to correlate with ADC_m_ parameter (25, 26). In the light of these previous studies, our findings indicated lower cell density of the tumors at the first time point measured.

According to our finding, non-Gaussian DWI models provided better fit to DWI data than the most commonly applied mono-exponential model, which is in line with previous findings (6, 16, 21, 27). The use of high b-values and non-Gaussian DWI models for early therapy response evaluation has demonstrated promising results in human brain tumor (28), colon cancer mouse model (29), and glioma mouse model (30). In a study by Hoff and co-workers (30), fast diffusion component of the bi-exponential model had the largest percent change from baseline in glioma mouse model, suggesting a role of non-Gaussian DWI models for prediction of early therapy response.

Several recent pre-clinical (31–33) and clinical (34–36) studies demonstrated promising results for IVIM derived parameters especially in organs with high perfusion, such as liver or kidney. In these organs the intravoxel incoherent motion present with relatively larger contribution to signal decay. The perfusion fraction was shown to be in the range of > 20-30%, being substantially more than that observed in brain, for example (37). In the current study, the averaged median perfusion fraction value was 9%, thus, being similar to human brain. Due to relatively low contribution of the intravoxel incoherent motion fraction to the measured signal, it is questionable how accurately a least square fitting procedure performed independently for each voxel (in a presence of measurement and physiological noise) can evaluated such small exponential component of the bi-exponential model. Despite preventing local minima in the fitting procedure, the repeatability of IVIM derived parameters (bi-exponential model for low b-values) was low. Coefficient of repeatability (expressed in % of averaged median values) for f_p_, D_p_ and D_f_ were 491.8%, 399.1% and 163.2%, respectively. Similar to our study, IVIM derived parameters using least square fitting have shown low reproducibility in human liver (38). The small contribution of “pseudodiffusion” component (f_p_, D_p_) to the final fitting residuals during least square fitting procedure, calls into question the validity of IVIM parameters that are estimated using least square fitting procedure for organs with small “pseudodiffusion” component (39–41). Efforts to increase fitting robustness of the bi-exponential model resulted into wider use of “segmented analysis” (32, 42) where each exponential component is being fitted individually in subsequent fashion. However, it should be noted that the resulting fitting residual is likely to be higher for “segmented analysis” than for “simultaneous” least square fitting of the bi-exponential model. Information content and bias of “segmented analysis” in comparison with other mathematical models remains to be established. Orton and the co-workers (43) have proposed the use of a Bayesian approach for improved estimation accuracy of IVIM parameters. Bayesian approach shrinks the distribution of parameters and “moves” outliers closer to the central distribution. Despite very promising results (36), this approach might not be applicable for cases with limited number of fitting voxels with large physiological voxel heterogeneity. Bayesian approach is a balance between improving quality of parametric maps and suppressing heterogeneity. Other possible approach is the use of neighborhood information during the fitting procedure (44).

Deviation of the DWI signal from mono-exponential decay at low b-values is, according to IVIM theory (6), due to intravoxel incoherent motion associated with capillary perfusion. “Perfusion fraction” (f_p_ in eq. 4) was proposed to reflex fractional volume (%) of capillary blood flow while “pseudodiffusion” (D_p_ in eq. 4) probably relates to blood velocity. Biological reasons for non-Gaussian DWI signal decay at high b-values remains open despite several proposed theories (7, 27, 45, 46). In the current study, the highest used b-value was 2000 s/mm^2^, which could have resulted in a less accurate estimation of slow diffusion component. Nevertheless, in vast majority of voxels non-Gaussian models provided better fit to DWI obtained using b-values up to 2000 s/mm^2^. As shown in prior studies (47, 48) of DWI models functions for PCa, that b-value distribution and DWI acquisition parameters may contribute to the fitting performance, and exploration of these factors is left for future studies.

Our study is limited by relatively small sample size. Furthermore, no attempts to histologically validate our findings with cell density have been made. Thus, further studies are needed to better investigate correlation of parameters derived from non-Gaussian DWI models with histopathological markers. Signal intensities differed systematically in the second DWI examination of the same tumor performed approximately 60 minutes after the first examination. This systematic bias in repeated DWI examinations has an effect on the estimation of repeatability. The confidence interval and coefficient of repeatability values between the repeated DWI examinations performed on week 4 are likely worse than those between weeks 1−4. Thus, the presented repeatability values should be regarded as the worst estimates due to systematic bias caused by DWI signal differences between the repeated DWI examinations. A potential cause of the bias is a temperature drop of the tumor (not mouse core temperature) in the second repeated DWI examination. Nevertheless, it is beyond the scope of the current study to fully explore an effect of temperature and anesthesia (26) on DWI decay curve derived parameters.

In conclusion, we have evaluated four different mathematical models for DWI of PC-3 prostate cancer cell derived xenografts in mice. Significant changes in the fitted parameters were present during tumor progression potentially due to increased cell density in later stages. The “pseudodiffusion” component in the analyzed tumors was shown to be less than 10% of the bi-exponential model. Due to low repeatability of the bi-exponential model parameters derived from low and high b-values DWI data using independent least square fitting on a voxel level, a degree of caution should be applied if these parameters are used for cancer characterization and therapy response monitoring. On the other hand, mono-exponential, stretched exponential, and kurtosis models shown high information content and robust repeatability.

## Data Availability Statement

The original contributions presented in the study are included in the article/**Supplementary Material**. Further inquiries can be directed to the corresponding author.

## Ethics Statement

The animal study was reviewed and approved by Turku Center for Disease Modeling, University of Turku, Turku, Finland.

## Author Contributions

All authors listed have made a substantial, direct, and intellectual contribution to the work and approved it for publication. Specifically, Conceptualization: HaM, AR, TL, IJ. Data curation: All authors. Funding acquisition: HA, TL, IJ. Investigation: HaL, HeL, HV, MP, TL. Methodology: All authors. Project administration: AR, TL, IJ. Resources: AR, MP, TL, IJ. Software and statistical analyses: HaM, IJ. Supervision: TL, IJ. Validation: HaM, IJ, Visualization: HaM, IJ. Writing – original draft: HaM, IJ. Writing – review & editing: HaM, AR, TL, IJ.

## Funding

This study was financially supported by grants Sigrid Jusélius Foundation (HaM, HA, and IJ), Finnish Cultural Foundation (HM and IJ), Finnish Cancer Society (IJ), Orion Research Foundation (HaM), Instrumentarium Research Foundation (IJ), Academy of Finland (AR and TL), Turku University Hospital (HA and IJ), TYKS-SAPA research fund (HA and IJ), Turku University Foundation (HaM and IJ), and University of Eastern Finland strategic funding (TL and HL).

## Conflict of Interest

The authors declare that the research was conducted in the absence of any commercial or financial relationships that could be construed as a potential conflict of interest.
