# Supplementary material for: Statistical Evaluation of Different Mathematical Models for Diffusion Weighted Imaging of Prostate Cancer Xenografts in Mice
Source: Front Oncol. 2021 May 26;11:583921. doi: 10.3389/fonc.2021.583921 (PMC8188898; doi:10.3389/fonc.2021.583921)
Supplement: Supplementary file 1 [file DataSheet_1.pdf]

Statistical evaluation of different mathematical models for diffusion weighted imaging of prostate cancer xenografts in mice

Supplementary Material

Low b-value DWI data (Figures S1-S10):

T2-weighted image fused with parametric maps of ADC_m, ADC_s, α, ADC_k, K, f_p, D_p, and D_f parameters are shown. The parametric maps are scaled as follows: ADC_m min-max: 0-2.0 $\mu m^2/ms$, ADC_s min-max: 0-1.5 $\mu m^2/ms$, α min-max: 0.6-1.0, ADC_k min-max: 0-2.0 $\mu m^2/ms$, K (E1-5), min-max: 0-3.0, f_p, min-max: 0-0.4, D_p min-max: 0-40.0 $\mu m^2/ms$, D_f min-max: 0-2.0 $\mu m^2/ms$.

High b-value DWI data (Figures S11-S20):

T2-weighted image fused with parametric maps of ADC_m, ADC_s , α, ADC_k, K, f_p, D_p, and D_f parameters are shown. The parametric maps are scaled as follows: ADC_m min-max: 0-2.0 $\mu m^2/ms$, ADC_s min-max: 0-1.5 $\mu m^2/ms$, α min-max: 0.6-1.0, ADC_k min-max: 0-2.0 $\mu m^2/ms$, K, min-max: 0-1.5, f_f min-max: 0-1.0, D_f min-max: 0-5.0 $\mu m^2/ms$, D_s min-max: 0-0.9 $\mu m^2/ms$.

Figure S1

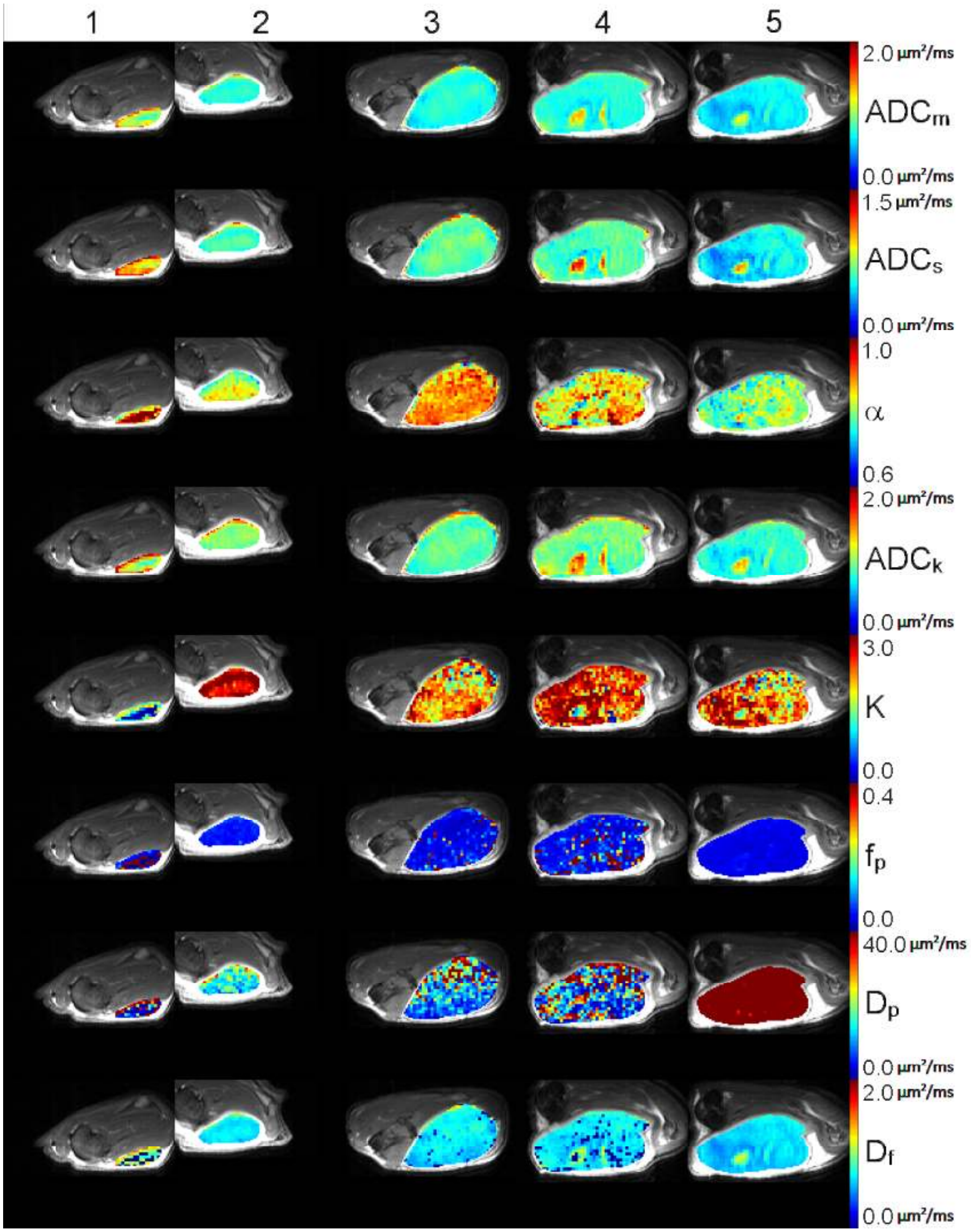

1
2
3
4
5
2.0 µm²/ms
ADCm
0.0 µm²/ms
1.5 µm²/ms
ADCs
0.0 µm²/ms
1.0
α
0.6
2.0 µm²/ms
ADCk
0.0 µm²/ms
3.0
K
0.0
0.4
fp
0.0
40.0 µm²/ms
Dp
0.0 µm²/ms
2.0 µm²/ms
Df
0.0 µm²/ms

Figure S2

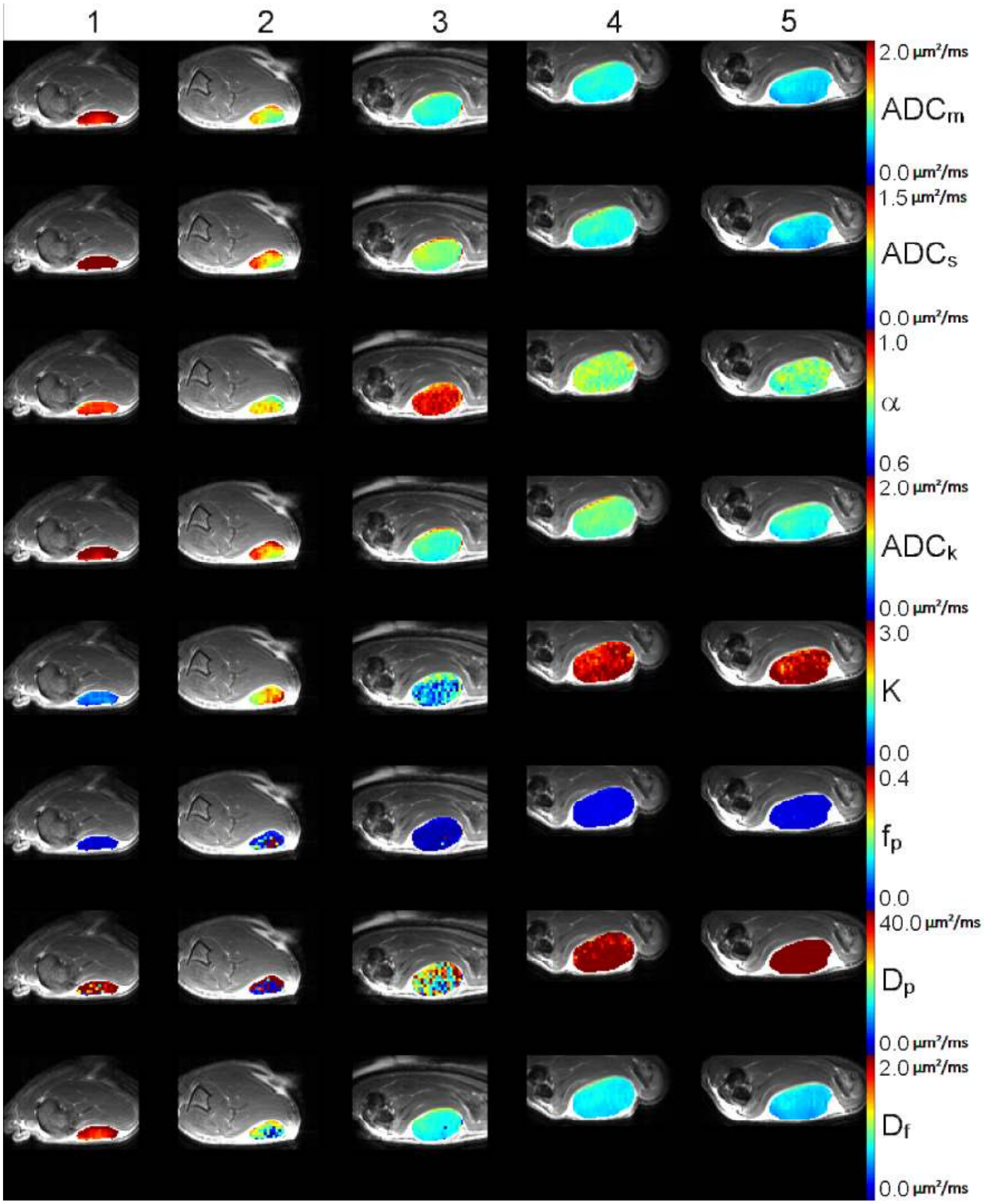
1
2
3
4
5
2.0 µm²/ms
ADC$_m$
0.0 µm²/ms
1.5 µm²/ms
ADC$_s$
0.0 µm²/ms
1.0
α
0.6
2.0 µm²/ms
ADC$_k$
0.0 µm²/ms
3.0
K
0.0
0.4
f$_p$
0.0
40.0 µm²/ms
D$_p$
0.0 µm²/ms
2.0 µm²/ms
D$_f$
0.0 µm²/ms

Figure S3

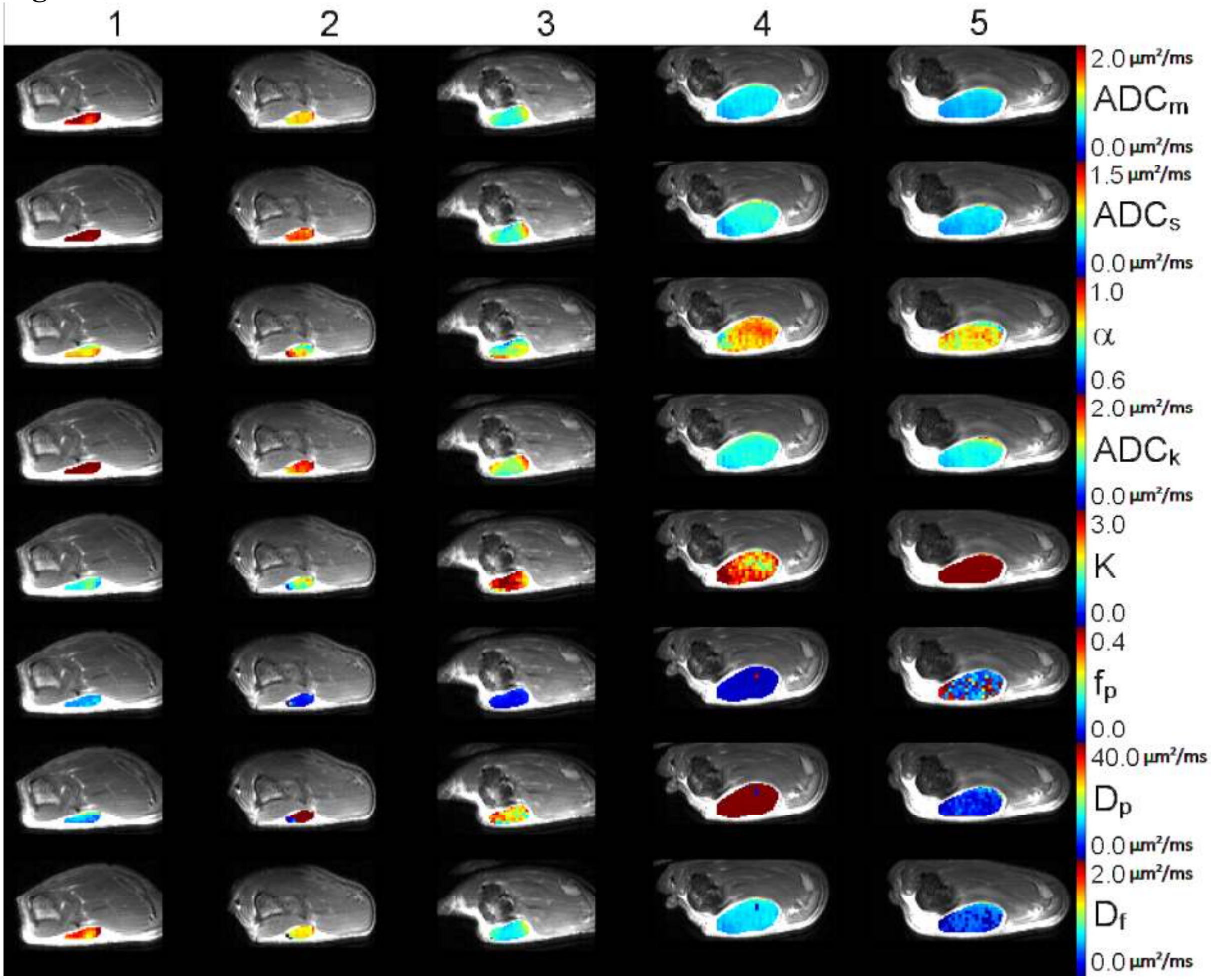
1
2
3
4
5
2.0 μm²/ms
ADCm
0.0 μm²/ms
1.5 μm²/ms
ADCs
0.0 μm²/ms
1.0
α
0.6
2.0 μm²/ms
ADCk
0.0 μm²/ms
3.0
K
0.0
0.4
fp
0.0
40.0 μm²/ms
Dp
0.0 μm²/ms
2.0 μm²/ms
Df
0.0 μm²/ms

Figure S4

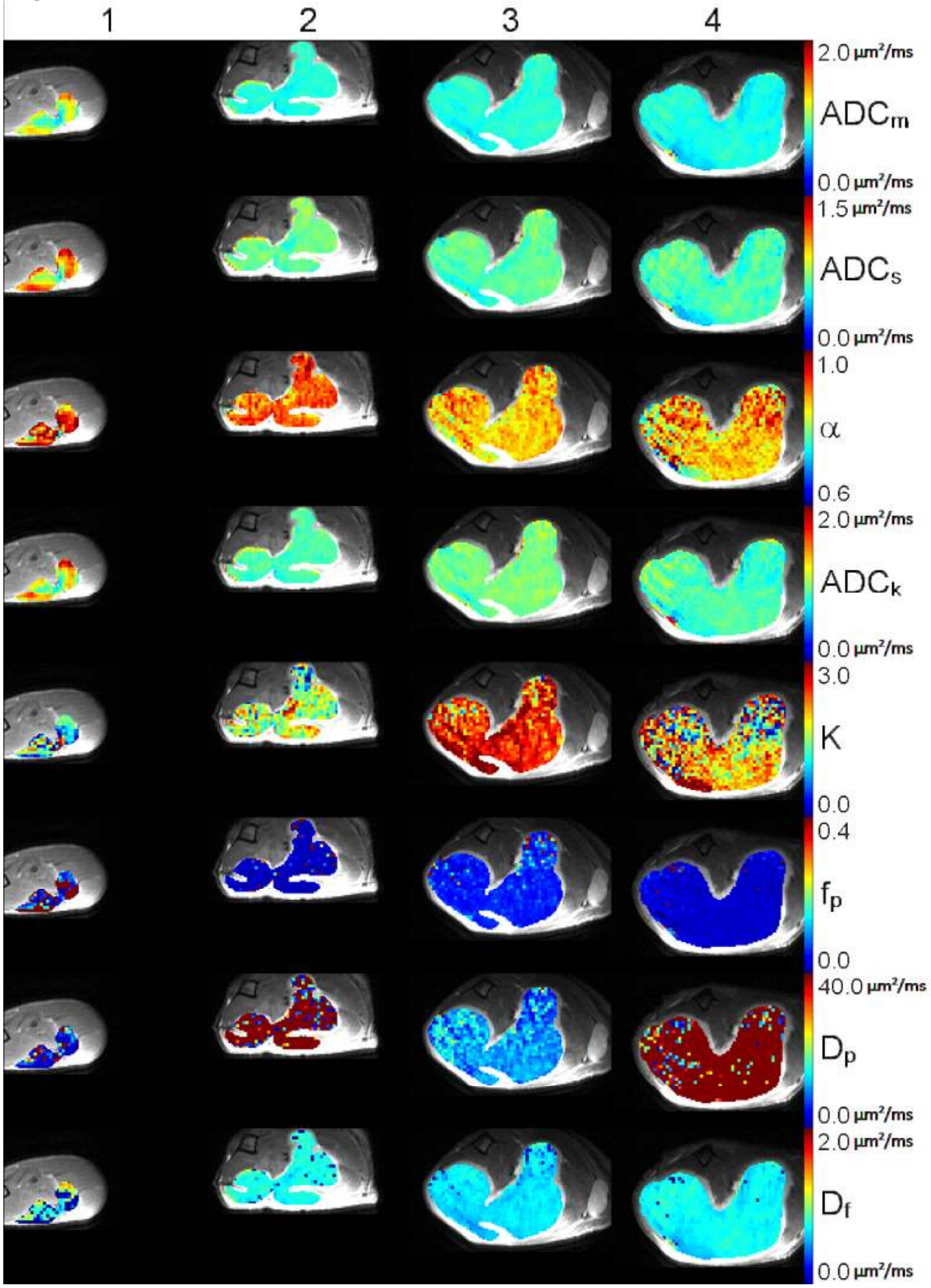
1
2
3
4
2.0 µm²/ms
ADCm
0.0 µm²/ms
1.5 µm²/ms
ADCs
0.0 µm²/ms
1.0
α
0.6
2.0 µm²/ms
ADCk
0.0 µm²/ms
3.0
K
0.0
0.4
fp
0.0
40.0 µm²/ms
Dp
0.0 µm²/ms
2.0 µm²/ms
Df
0.0 µm²/ms

Figure S5

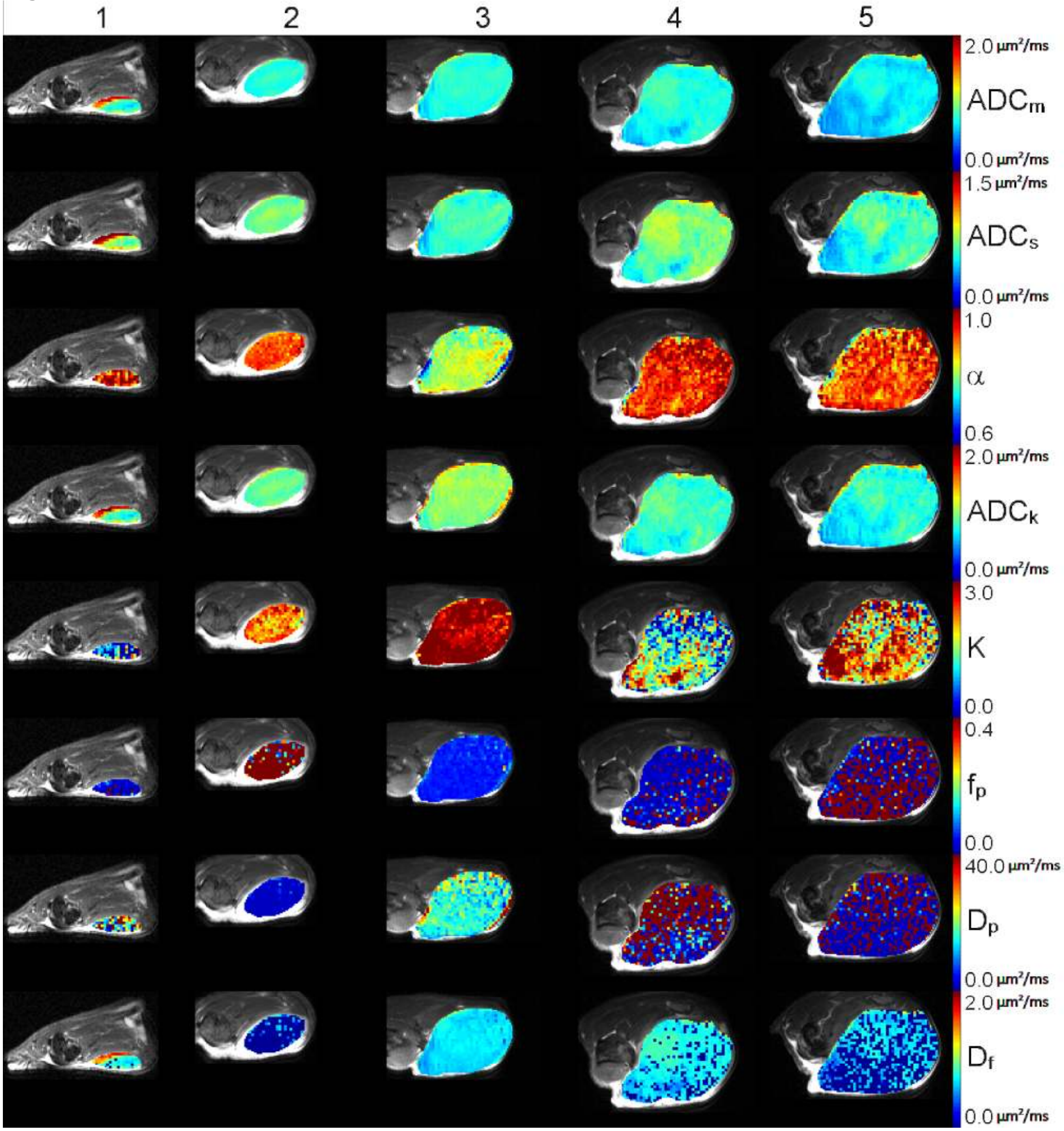
1
2
3
4
5
2.0 µm²/ms
ADCm
0.0 µm²/ms
1.5 µm²/ms
ADCs
0.0 µm²/ms
1.0
α
0.6
2.0 µm²/ms
ADCk
0.0 µm²/ms
3.0
K
0.0
0.4
fp
0.0
40.0 µm²/ms
Dp
0.0 µm²/ms
2.0 µm²/ms
Df
0.0 µm²/ms

Figure S6

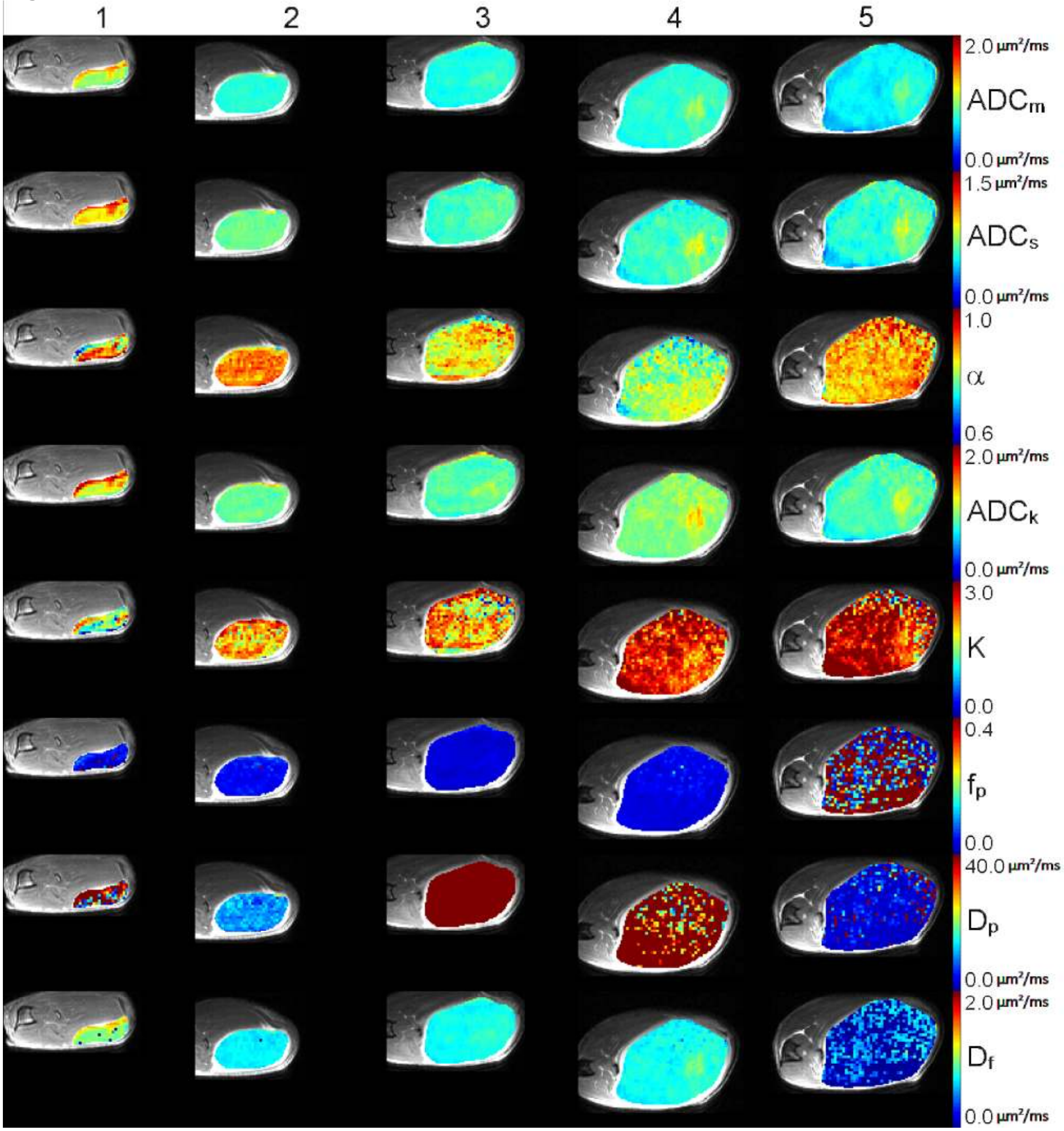
1
2
3
4
5
2.0 µm²/ms
ADCm
0.0 µm²/ms
1.5 µm²/ms
ADCs
0.0 µm²/ms
1.0
α
0.6
2.0 µm²/ms
ADCk
0.0 µm²/ms
3.0
K
0.0
0.4
fp
0.0
40.0 µm²/ms
Dp
0.0 µm²/ms
2.0 µm²/ms
Df
0.0 µm²/ms

Figure S7

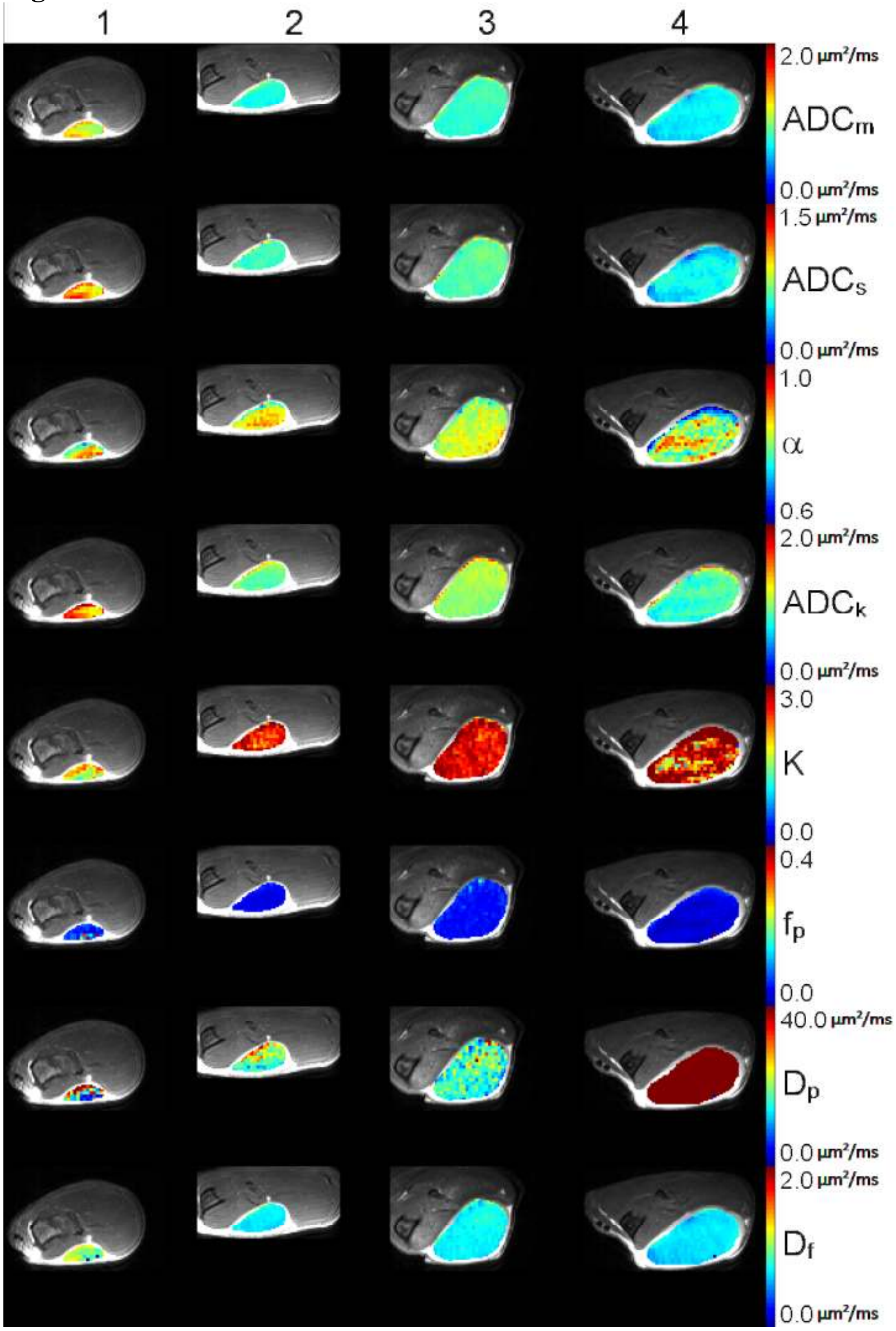

1
2
3
4
2.0 μm²/ms
ADCm
0.0 μm²/ms
1.5 μm²/ms
ADCs
0.0 μm²/ms
1.0
α
0.6
2.0 μm²/ms
ADCk
0.0 μm²/ms
3.0
K
0.0
0.4
fp
0.0
40.0 μm²/ms
Dp
0.0 μm²/ms
2.0 μm²/ms
Df
0.0 μm²/ms

Figure S8

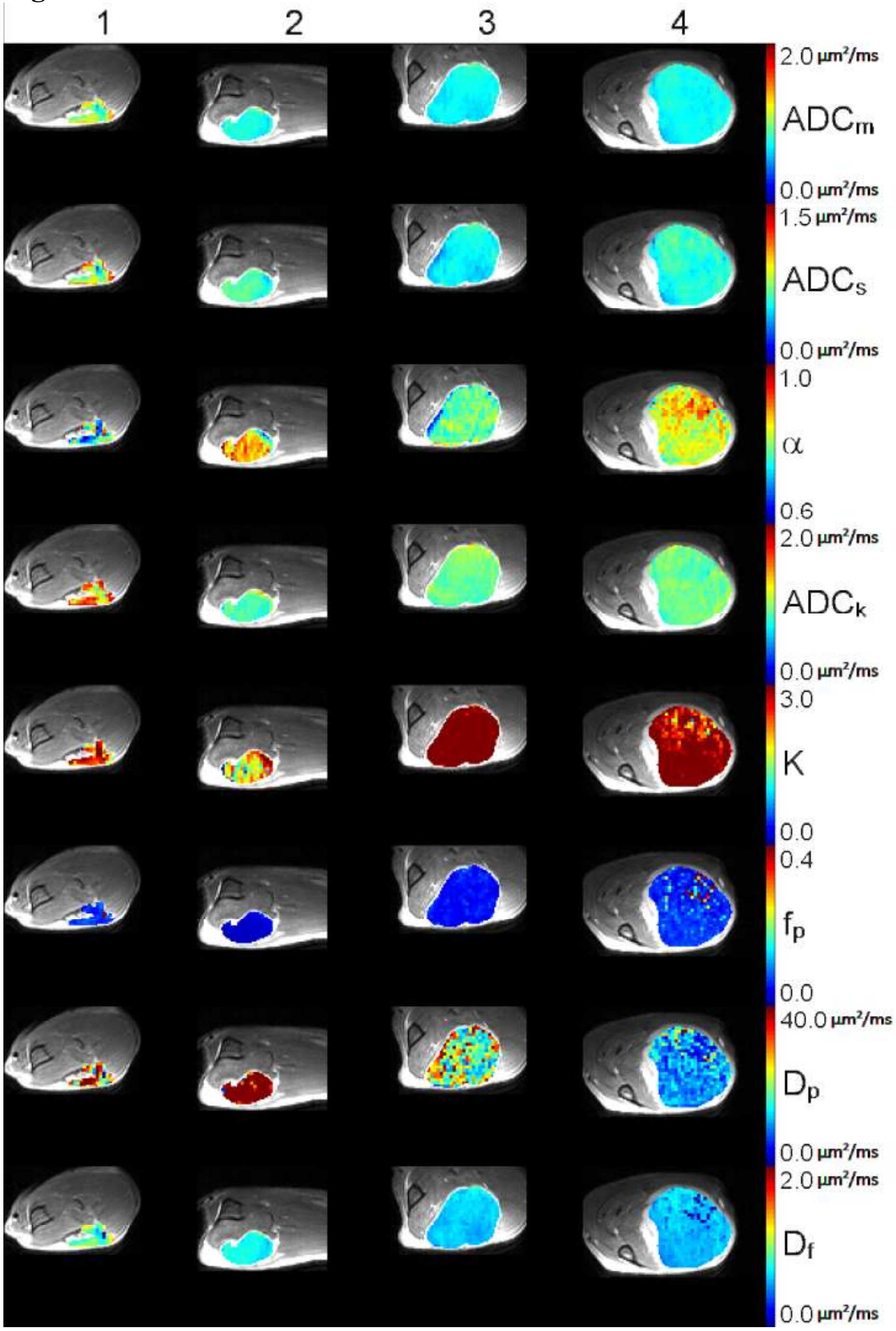
1
2
3
4
2.0 μm²/ms
ADCm
0.0 μm²/ms
1.5 μm²/ms
ADCs
0.0 μm²/ms
1.0
α
0.6
2.0 μm²/ms
ADCk
0.0 μm²/ms
3.0
K
0.0
0.4
fp
0.0
40.0 μm²/ms
Dp
0.0 μm²/ms
2.0 μm²/ms
Df
0.0 μm²/ms

Figure S9

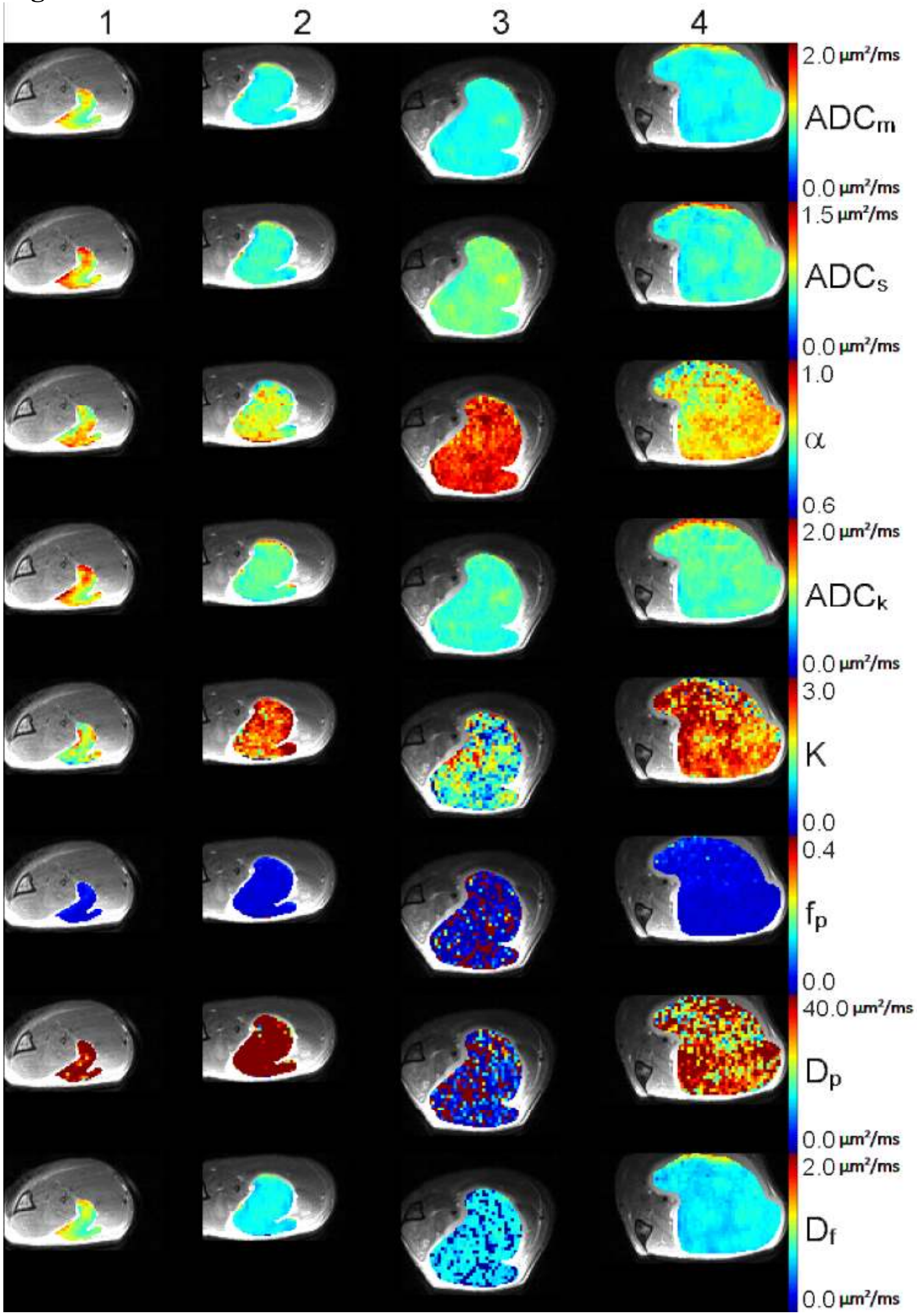

1
2
3
4
2.0 µm²/ms
ADCm
0.0 µm²/ms
1.5 µm²/ms
ADCs
0.0 µm²/ms
1.0
α
0.6
2.0 µm²/ms
ADCk
0.0 µm²/ms
3.0
K
0.0
0.4
fp
0.0
40.0 µm²/ms
Dp
0.0 µm²/ms
2.0 µm²/ms
Df
0.0 µm²/ms

Figure S10

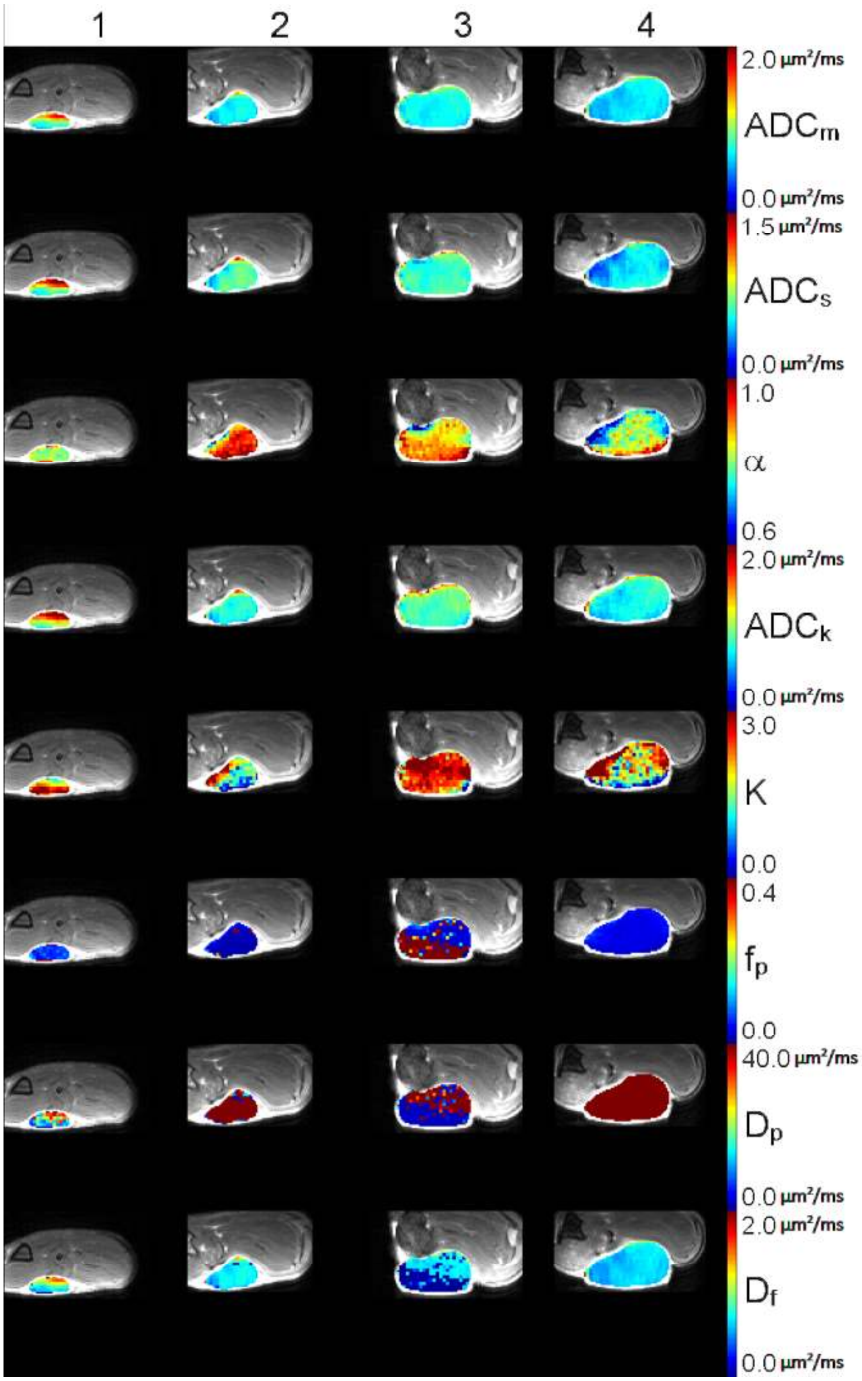
1
2
3
4
2.0 µm²/ms
ADCm
0.0 µm²/ms
1.5 µm²/ms
ADCs
0.0 µm²/ms
1.0
α
0.6
2.0 µm²/ms
ADCk
0.0 µm²/ms
3.0
K
0.0
0.4
fp
0.0
40.0 µm²/ms
Dp
0.0 µm²/ms
2.0 µm²/ms
Df
0.0 µm²/ms

Figure S11

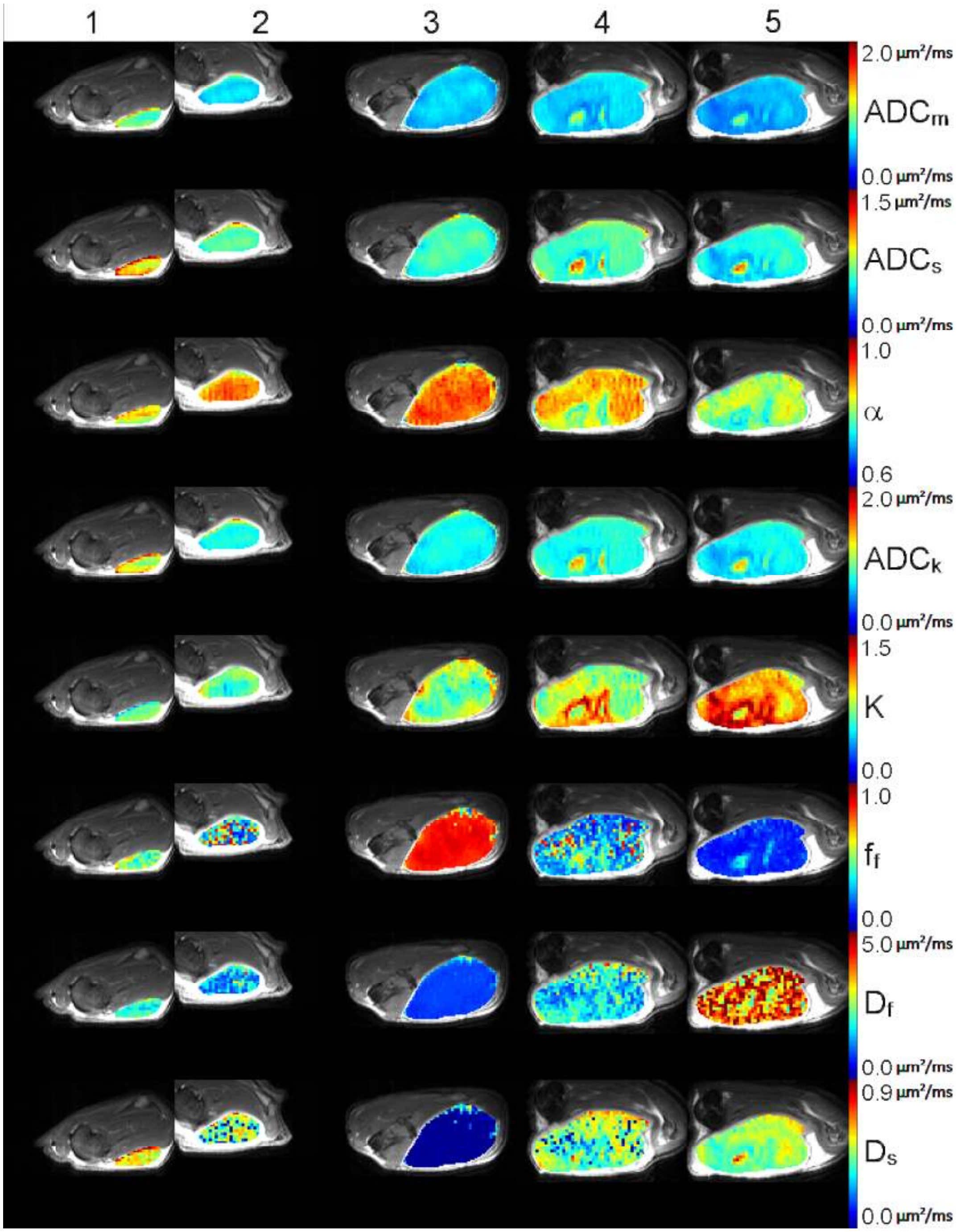
1
2
3
4
5
2.0 µm²/ms
ADCm
0.0 µm²/ms
1.5 µm²/ms
ADCs
0.0 µm²/ms
1.0
α
0.6
2.0 µm²/ms
ADCk
0.0 µm²/ms
1.5
K
0.0
1.0
ff
0.0
5.0 µm²/ms
Df
0.0 µm²/ms
0.9 µm²/ms
Ds
0.0 µm²/ms

Figure S12

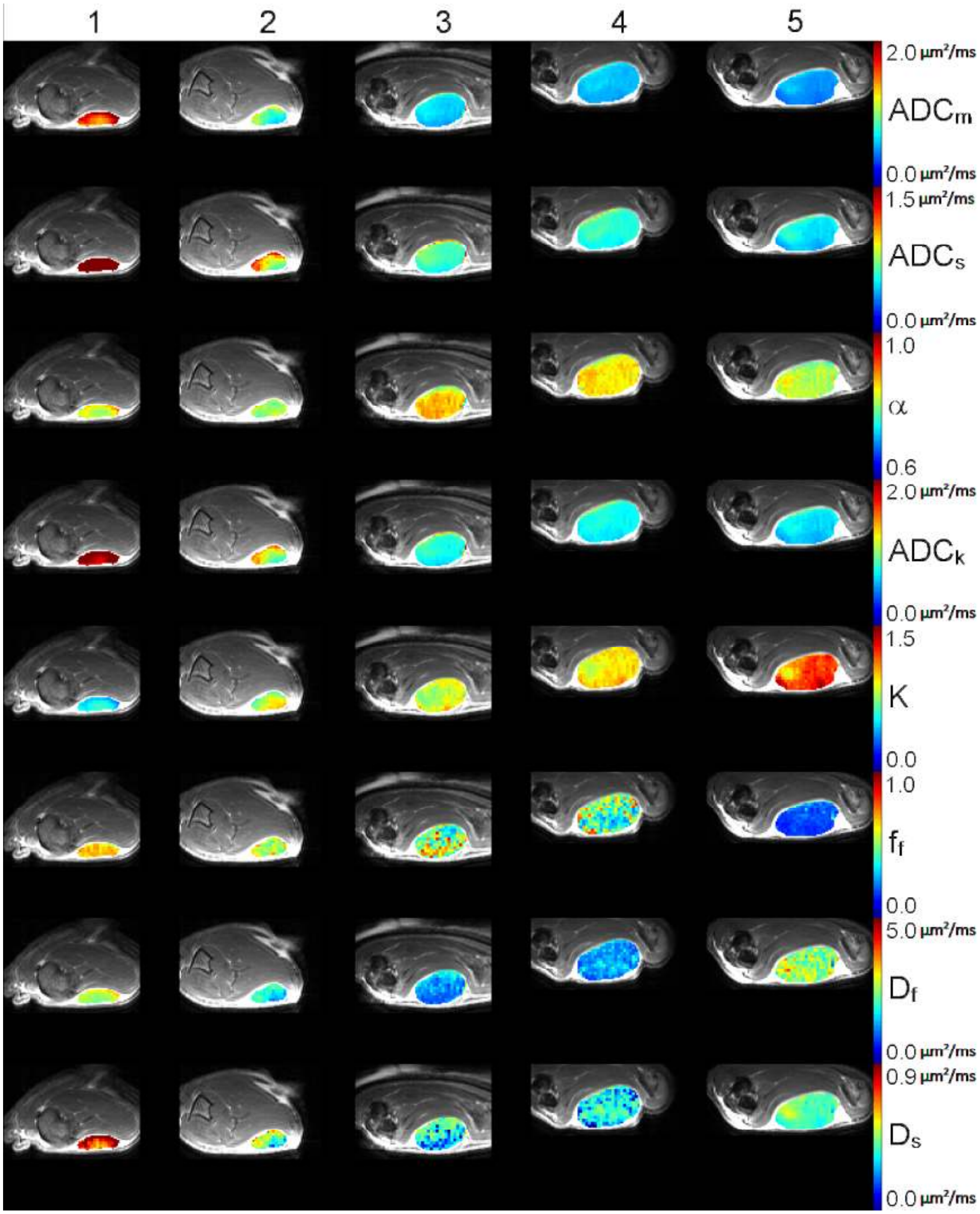
1
2
3
4
5
2.0 μm²/ms
ADCm
0.0 μm²/ms
1.5 μm²/ms
ADCs
0.0 μm²/ms
1.0
α
0.6
2.0 μm²/ms
ADCk
0.0 μm²/ms
1.5
K
0.0
1.0
ff
0.0
5.0 μm²/ms
Df
0.0 μm²/ms
0.9 μm²/ms
Ds
0.0 μm²/ms

Figure S13

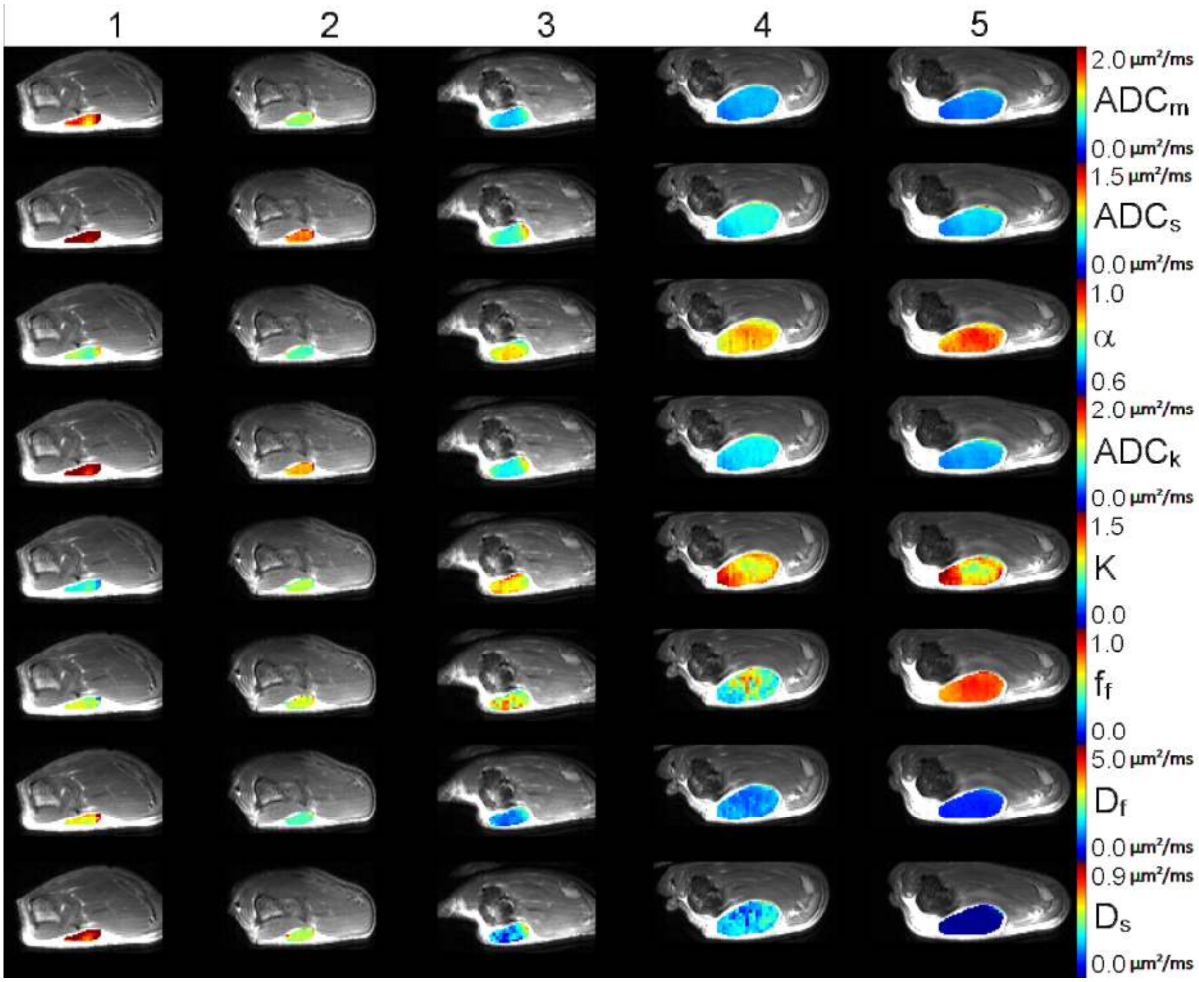

1
2
3
4
5
2.0 μm²/ms
ADCm
0.0 μm²/ms
1.5 μm²/ms
ADCs
0.0 μm²/ms
1.0
α
0.6
2.0 μm²/ms
ADCk
0.0 μm²/ms
1.5
K
0.0
1.0
ff
0.0
5.0 μm²/ms
Df
0.0 μm²/ms
0.9 μm²/ms
Ds
0.0 μm²/ms

Figure S14

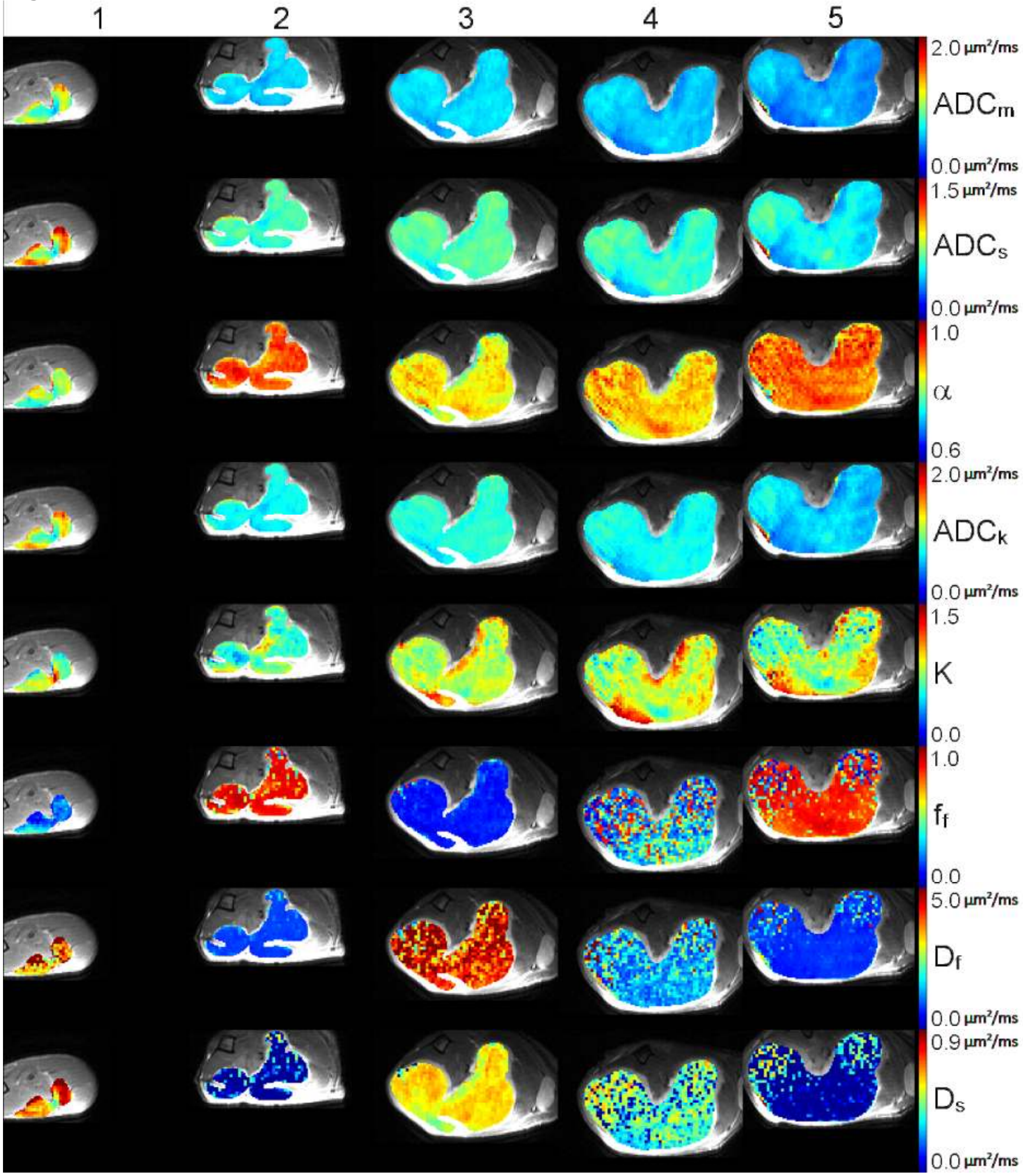

1
2
3
4
5
2.0 μm²/ms
ADCm
0.0 μm²/ms
1.5 μm²/ms
ADCs
0.0 μm²/ms
1.0
α
0.6
2.0 μm²/ms
ADCk
0.0 μm²/ms
1.5
K
0.0
1.0
ff
0.0
5.0 μm²/ms
Df
0.0 μm²/ms
0.9 μm²/ms
Ds
0.0 μm²/ms

Figure S15

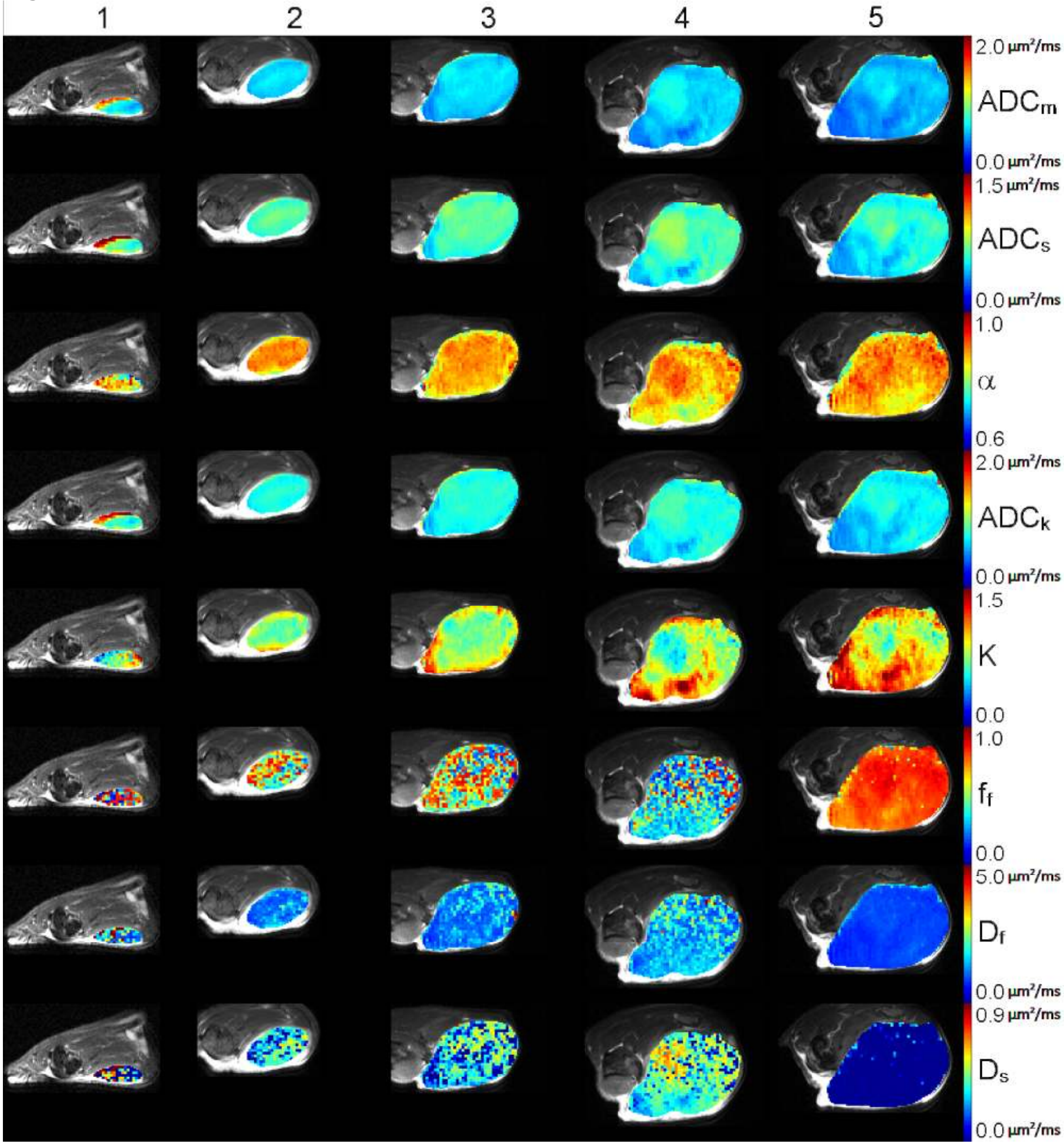
1
2
3
4
5
2.0 µm²/ms
ADCm
0.0 µm²/ms
1.5 µm²/ms
ADCs
0.0 µm²/ms
1.0
α
0.6
2.0 µm²/ms
ADCk
0.0 µm²/ms
1.5
K
0.0
1.0
ff
0.0
5.0 µm²/ms
Df
0.0 µm²/ms
0.9 µm²/ms
Ds
0.0 µm²/ms

Figure S16

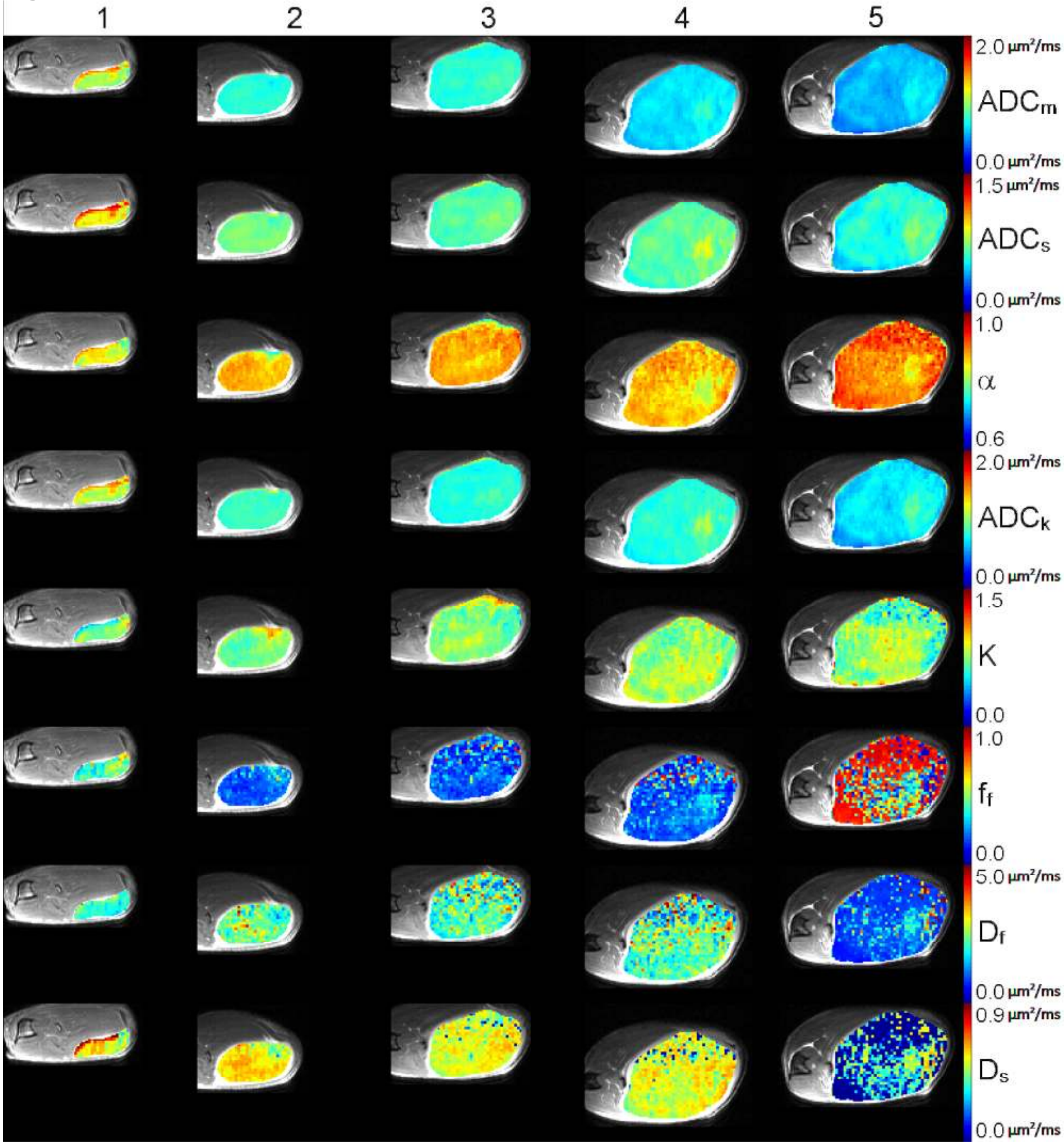

1
2
3
4
5
2.0 μm²/ms
ADCm
0.0 μm²/ms
1.5 μm²/ms
ADCs
0.0 μm²/ms
1.0
α
0.6
2.0 μm²/ms
ADCk
0.0 μm²/ms
1.5
K
0.0
1.0
ff
0.0
5.0 μm²/ms
Df
0.0 μm²/ms
0.9 μm²/ms
Ds
0.0 μm²/ms

Figure 17

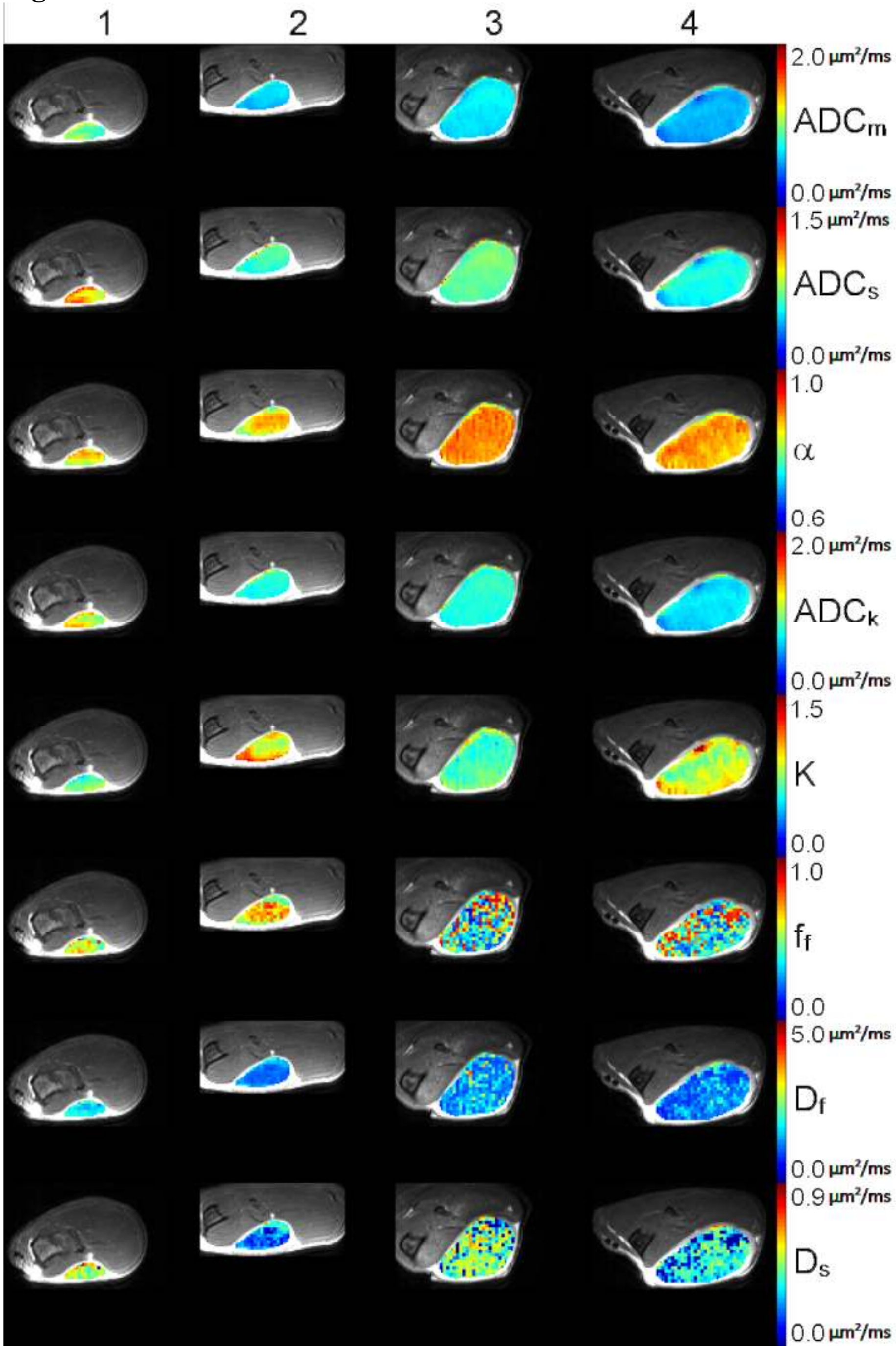

1
2
3
4
2.0 µm²/ms
ADCm
0.0 µm²/ms
1.5 µm²/ms
ADCs
0.0 µm²/ms
1.0
α
0.6
2.0 µm²/ms
ADCk
0.0 µm²/ms
1.5
K
0.0
1.0
ff
0.0
5.0 µm²/ms
Df
0.0 µm²/ms
0.9 µm²/ms
Ds
0.0 µm²/ms

Figure 18

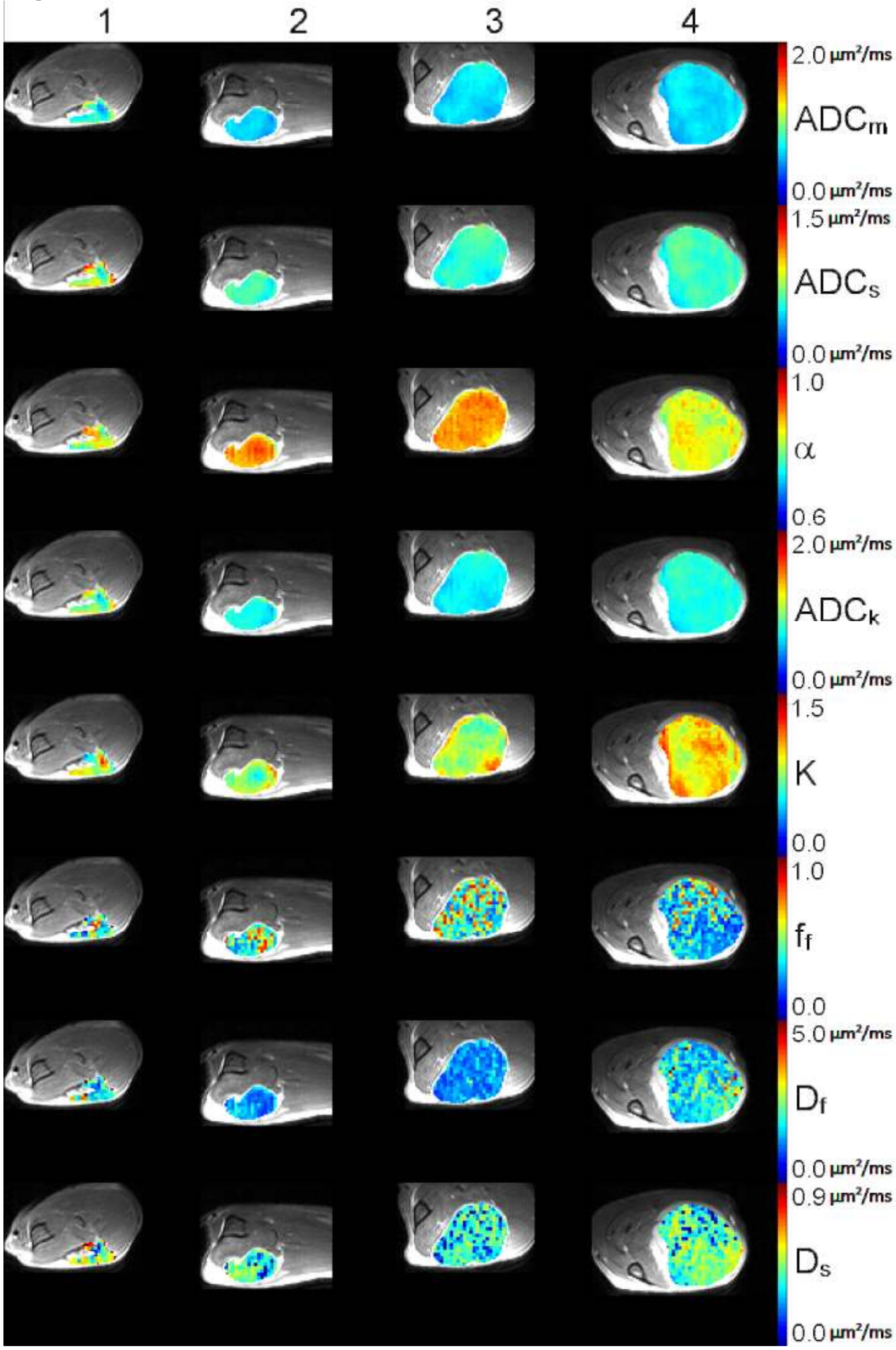

1
2
3
4
2.0 μm²/ms
ADCm
0.0 μm²/ms
1.5 μm²/ms
ADCs
0.0 μm²/ms
1.0
α
0.6
2.0 μm²/ms
ADCk
0.0 μm²/ms
1.5
K
0.0
1.0
ff
0.0
5.0 μm²/ms
Df
0.0 μm²/ms
0.9 μm²/ms
Ds
0.0 μm²/ms

Figure 19

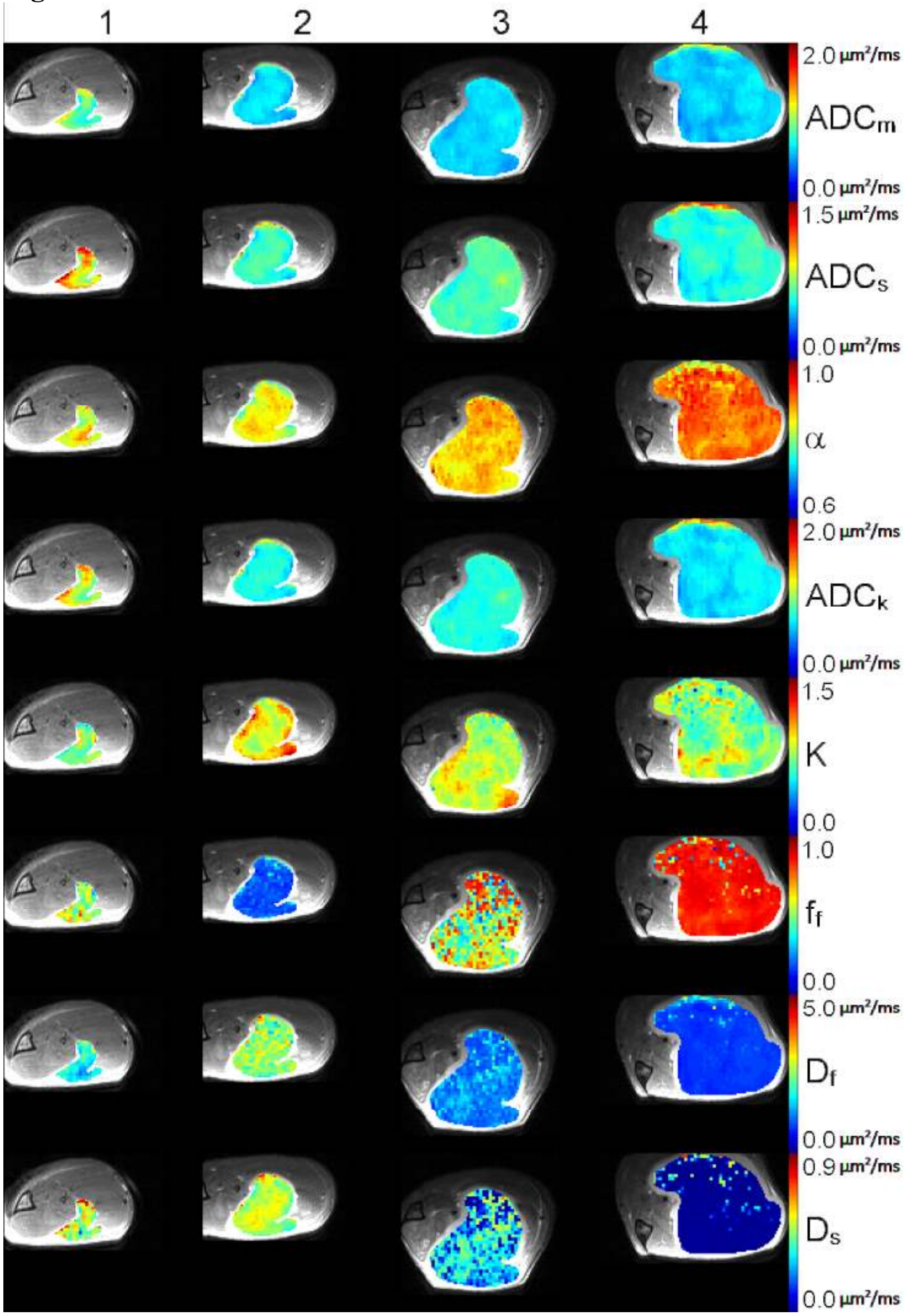
1
2
3
4
2.0 µm²/ms
ADCm
0.0 µm²/ms
1.5 µm²/ms
ADCs
0.0 µm²/ms
1.0
α
0.6
2.0 µm²/ms
ADCk
0.0 µm²/ms
1.5
K
0.0
1.0
ff
0.0
5.0 µm²/ms
Df
0.0 µm²/ms
0.9 µm²/ms
Ds
0.0 µm²/ms

Figure 20

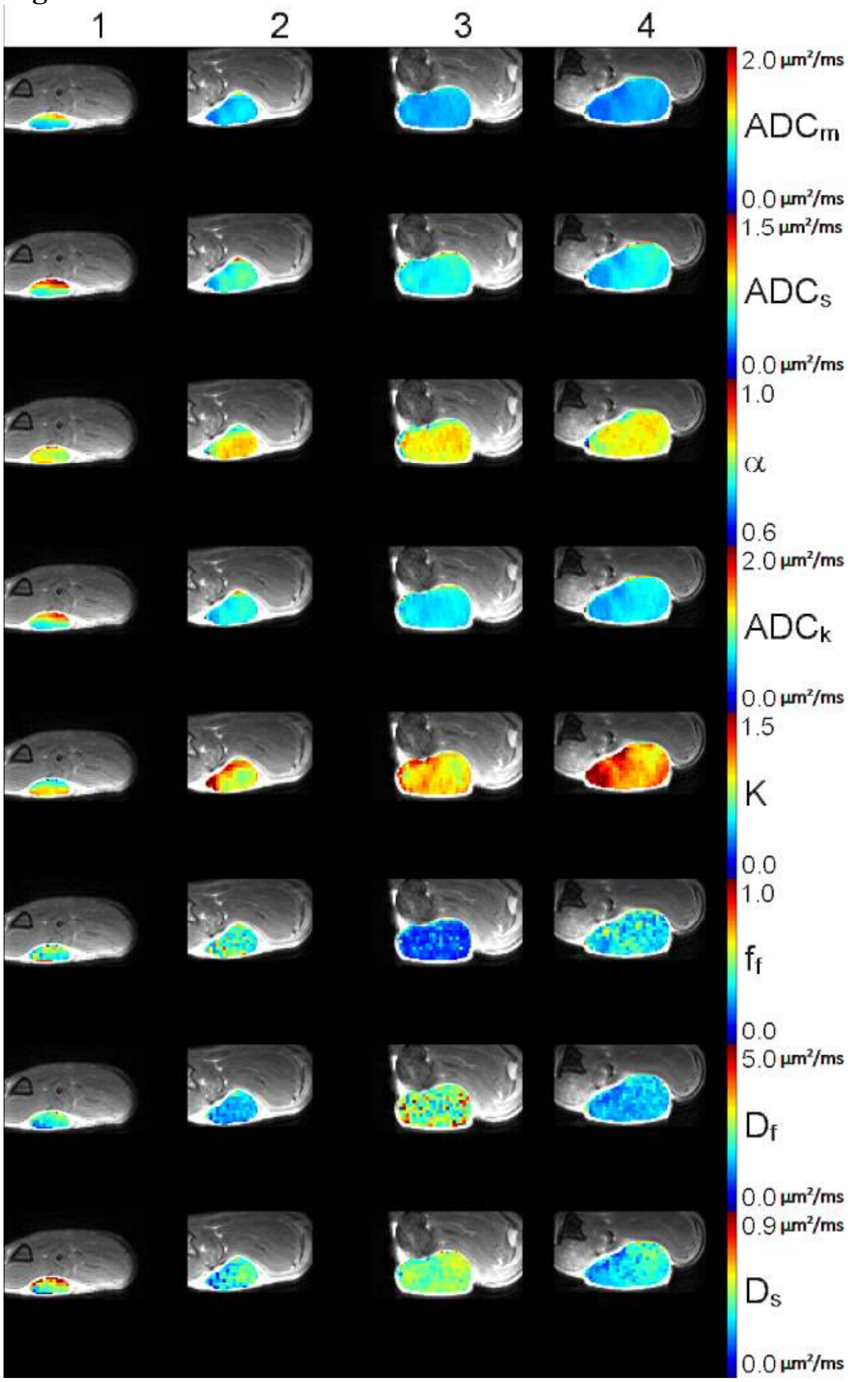

1
2
3
4
2.0 µm²/ms
ADCm
0.0 µm²/ms
1.5 µm²/ms
ADCs
0.0 µm²/ms
1.0
α
0.6
2.0 µm²/ms
ADCk
0.0 µm²/ms
1.5
K
0.0
1.0
ff
0.0
5.0 µm²/ms
Df
0.0 µm²/ms
0.9 µm²/ms
Ds
0.0 µm²/ms
